# Rice ragged stunt virus Pns10 induces mitochondrial-mediated apoptosis to promote viral infection in *Nilaparvata lugens* through disrupting the NlNDUFS1-NlPHB2 interaction

**DOI:** 10.1371/journal.ppat.1013415

**Published:** 2025-08-19

**Authors:** Lianshun Zheng, Shuai Fu, Ming Zeng, Liyan Li, Dan Wang, Shibo Gao, Yunge Zhang, Cui Zhang, Shifang Fei, Xuan Ye, Lele Chen, Qianhui Chen, Yaqin Wang, Xueping Zhou, Yan Xie, Boli Hu, Jianxiang Wu

**Affiliations:** 1 State Key Laboratory of Rice Biology and Breeding, Zhejiang Key Laboratory of Biology and Ecological Regulation of Crop Pathogens and Insects, Institute of Biotechnology, Zhejiang University, Hangzhou, Zhejiang, P.R. China; 2 Hainan Institute of Zhejiang University, Sanya, P.R. China; 3 Research Center for Life Sciences Computing, Zhejiang Lab, Hangzhou, China; 4 State Key Laboratory for Biology of Plant Diseases and Insect Pests, Institute of Plant Protection, Chinese Academy of Agricultural Sciences, Beijing, P.R. China; 5 MOA Key Laboratory of Animal Virology, Zhejiang University Center for Veterinary Sciences, Hangzhou, P.R. China; Instituto de Biología Molecular y Celular de Plantas (CSIC-Universidad Politécnica de Valencia), SPAIN

## Abstract

Apoptosis, a programmed cell death process, plays crucial roles in host antiviral response. Although there are many reports on the relationship between cell apoptosis and viral infection, the mechanisms underlying plant arbovirus-induced apoptosis in insect vectors remain largely unclear. Here, we reported that apoptosis promotes rice ragged stunt virus (RRSV) infection in *Nilaparvata lugens* (brown planthopper), and RRSV-encoded Pns10 protein can induce apoptosis in *N. lugens*. The Pns10 interacts with *N. lugens* NADH:ubiquinone oxidoreductase 75 kDa Fe-S protein 1(NlNDUFS1), a core subunit of mitochondrial complex I. Silencing of *NlNDUFS1* expression in *N. lugens* impaired mitochondrial complex I activity, decreasing ATP production and increasing mitochondrial ROS accumulation. This dysregulation triggers apoptosis to promote RRSV infection in *N. lugens*. Furthermore, RRSV Pns10 disrupts the interaction between NlNDUFS1 and NlProhibitin 2 (NlPHB2) in *N. lugens* to impair mitochondrial complex I activity, leading to a decrease of ATP production and an increase of mitochondrial ROS accumulation. The excessive accumulation of mitochondrial ROS causes genomic DNA fragmentation and apoptosis. Collectively, the findings presented here illuminate a novel mechanism by which a plant virus manipulates vector mitochondrial apoptosis to benefit viral infection, and offer insights for future transmission-blocking interventions.

## Author summary

Arbovirus vectors play a pivotal role in the transmission of crop virus diseases. While apoptosis serves as a critical antiviral defense, some viruses can hijack apoptosis pathway to promote their infection. However, the mechanisms underlying plant arbovirus-induced apoptosis in insect vectors remain largely unclear. Here, we find that a plant arbovirus, rice ragged stunt virus (RRSV), can induce apoptosis to promote its infection in *Nilaparvata lugens*. We find that RRSV-encoded Pns10 interacts with NlNDUFS1, an important component of mitochondrial complex I in *N. lugens*. The Pns10 impairs mitochondrial complex I activity in *N. lugens* through disrupting the NlNDUFS1 and NlPHB2 interaction. This impairment of mitochondrial complex I activity results in a significant decrease of ATP production and a significant increase of mitochondrial ROS accumulation, causing mitochondrial apoptosis in *N. lugens* to promote RRSV replication. Our research uncovers the molecular mechanism that RRSV induces mitochondrial apoptosis to promote its replication in *N. lugens* vector.

## Introduction

Apoptosis, a programmed cell death process, exists in all organisms and plays important roles in many life processes [1,2]. Apoptosis is characterized by cell shrinkage, chromatin condensation, nuclear fragmentation, membrane blebbing, and cell fragment division into membrane-bound vesicles (apoptotic bodies) that are absorbed by other cells and rapidly degraded by lysosomal enzymes [2–5]. Caspases, a family of cysteine proteases, have been reported to play crucial roles in initiating and executing apoptosis [6]. Apoptosis can be triggered by the intrinsic mitochondria-mediated pathway, extrinsic death receptor-mediated pathway, granzyme B-mediated pathway, and endoplasmic reticulum (ER)-mediated pathway [6,7]. In addition, loss of mitochondrial membrane potential is often considered as an early signal of mitochondria-mediated apoptosis, while Bcl-2 can prevent the mitochondria-mediated apoptosis [8,9].

Apoptosis plays important roles in antiviral defenses through elimination of virus-infected cells [10–12]. On the other hand, many viruses have evolved different mechanisms to inhibit host cell apoptosis to complete their replication cycles or directly or indirectly induce cell apoptosis to promote their proliferations and spread in their hosts [13,14]. For example, several recent reports have shown that some arboviruses can manipulate the apoptotic pathway in insect vectors to promote their infections [9,15]. However, to date, how arboviruses regulate apoptosis in their insect vectors remain to be unraveled.

Mitochondrial complex I, also known as NADH:ubiquinone oxidoreductase, is the first and largest enzyme involved in the mitochondrial respiratory chain. It catalyzes electron transfer from NADH to coenzyme Q10 to support ATP synthesis in mammalian mitochondria [16,17]. Mammalian mitochondrial complex I consists of 45 subunits. Of these 45 subunits, seven are encoded by mitochondrial genes, while others are encoded by nuclear genes [18]. NADH:ubiquinone oxidoreductase 75 kDa Fe-S protein 1 (NDUFS1) encoded by a nuclear gene is one of the core subunits of mitochondrial complex I. NDUFS1 contains three iron-sulfur clusters in the N-module which functions to bind and oxidize NADH [17,18]. It can catalyze the entry and efficient transfer of electrons within mitochondrial complex I and to promote the formation of supercomplexes of mitochondrial complex I and the ubiquinol-cytochrome c reductase complex (Complex III) [17,19]. In addition, NDUFS1 has been reported to regulate mitochondrial ATP and ROS productions, and to play important roles in the metabolic reprogramming, oxidative stress, and apoptosis in animals [20–22].

Rice ragged stunt virus (RRSV) is a member of the genus *Oryzavirus*, *Spinareoviridae* family, and is transmitted by *Nilaparvata lugens* in a persistent-propagative manner [23,24]. RRSV has been found in rice fields in Southern China, Japan, and many Southeast Asian countries, and often causes severe rice yield losses [24]. Our field surveys in recent years found that this viral disease is prevalent in rice fields in Hainan, Guangxi, and Guangdong provinces of China. RRSV virions are double-layered, icosahedral particles with 75–80 nm in diameter. It contains 10 linear double-stranded genomic RNA segments (S1–S10) that encodes a total of 11 proteins: eight structural proteins (i.e., P1, P2, P3, P4a, P4b, P5, P8b, and P9) and three nonstructural proteins (i.e., Pns 6, Pns 7, and Pns 10) [23,25]. The S10-encoded Pns10 has been reported to have ATPase activity and to promote viral nucleic acid packaging and double-layered capsid assembly [24,26]. In 2016, Huang and colleagues reported that RRSV Pns10 interacts with oligomycin-sensitivity conferral protein (OSCP) on the mitochondrial inner membrane in *N. lugens*, and silencing of *OSCP* expression significantly inhibits the accumulation level of RRSV in infected *N. lugens* [26]. RRSV infection has also been shown to induce apoptosis in *N. lugens* salivary gland cells [27]. In addition, Huang and colleagues identified five caspase genes (*caspase1a*, *caspase1b*, *caspase1c*, *caspase8*, and *caspaseNc*) in *N. lugens* [27].

Here, we provide evidences to show that RRSV infection activates mitochondrion-dependent apoptosis to promote RRSV infection in *N. lugens*. The RRSV-encoded Pns10 interacts with NlNDUFS1, an important component of mitochondrial complex I in *N. lugens*, to disrupt the NlNDUFS1 and NlPHB2 interaction, resulting in a significant reduce of mitochondrial complex I activity. In addition, the reduction of mitochondrial complex I activity decreases ATP production, and increases mitochondrial ROS accumulation, thereby activating cell apoptosis to promote RRSV infection in *N. lugens*.

## Results

### RRSV infection induces apoptosis in *N. lugens*

**Fig 1:**
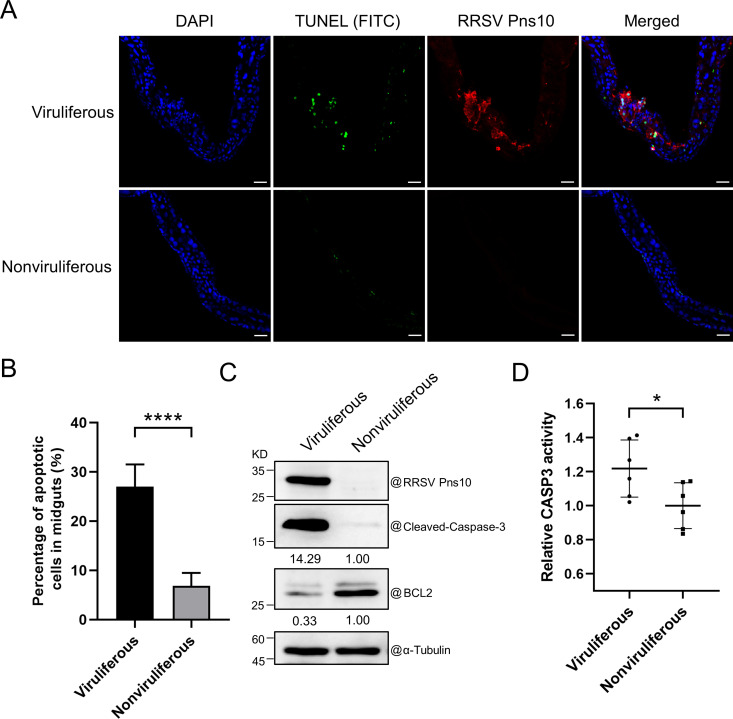
RRSV infection induces apoptosis in *N. lugens.* **(A)** Confocal images showing increased TUNEL-positive cells (FITC, green) in viruliferous *N. lugens* midguts, co-stained with Pns10 (Dylight 549, red) and nuclei (DAPI, blue). Scale bar = 50 μm. **(B)** The percentage of apoptotic cells was determined based on the number of TUNEL-positive cells over the number of DAPI-positive cells in midguts. The values are the means ± SDs (n = 5), determined using the Student’s *t* test. **** indicates *P* < 0.0001. **(C)** Analysis of apoptotic response in RRSV viruliferous or nonviruliferous *N. lugens* through Western blot assays using a cleaved-caspase-3 or a BCL2 antibody. An α-Tubulin antibody is used to show sample loadings. **(D)** Analysis of CASP3 activity in RRSV viruliferous or nonviruliferous *N. lugens* using a caspase 3 activity assay kit. The values are the means ± SDs (n = 6), determined using the Student’s *t t*est. * indicates **P* *<* *0.05.

To explore whether RRSV infection causes apoptosis in *N. lugens*, we sampled *N. lugens* midguts at 4 days post virus feeding (dpvf) and analyzed them through TUNEL staining. Compared with midguts from nonviruliferous *N. lugens*, more cells in the midguts from RRSV viruliferous *N. lugens* showed positive TUNEL staining signal (green) under the confocal microscope (Fig 1A and 1B). Furthermore, the apoptotic protein marker cleaved-caspase-3 and the degradation of anti-apoptotic protein BCL2 were detected in viruliferous and nonviruliferous *N. lugens* by Western blot assay. Western blot assay result showed that compared with those in nonviruliferous *N. lugens*, the accumulation level of cleaved-caspase-3 significantly increased, while the accumulation level of BCL2 was significantly decreased in viruliferous *N. lugens* at 6 dpvf (Fig 1C)*.* Additionally, through analysis of caspase 3 (CASP3) activity in viruliferous *N. lugens* at 10 dpvf or in nonviruliferous control, we found that CASP3 activity in viruliferous *N. lugens* was significantly increased (Fig 1D), indicating that RRSV infection does induce apoptosis in *N. lugens.*

### Apoptosis promotes RRSV accumulation in *N. lugens*

**Fig 2:**
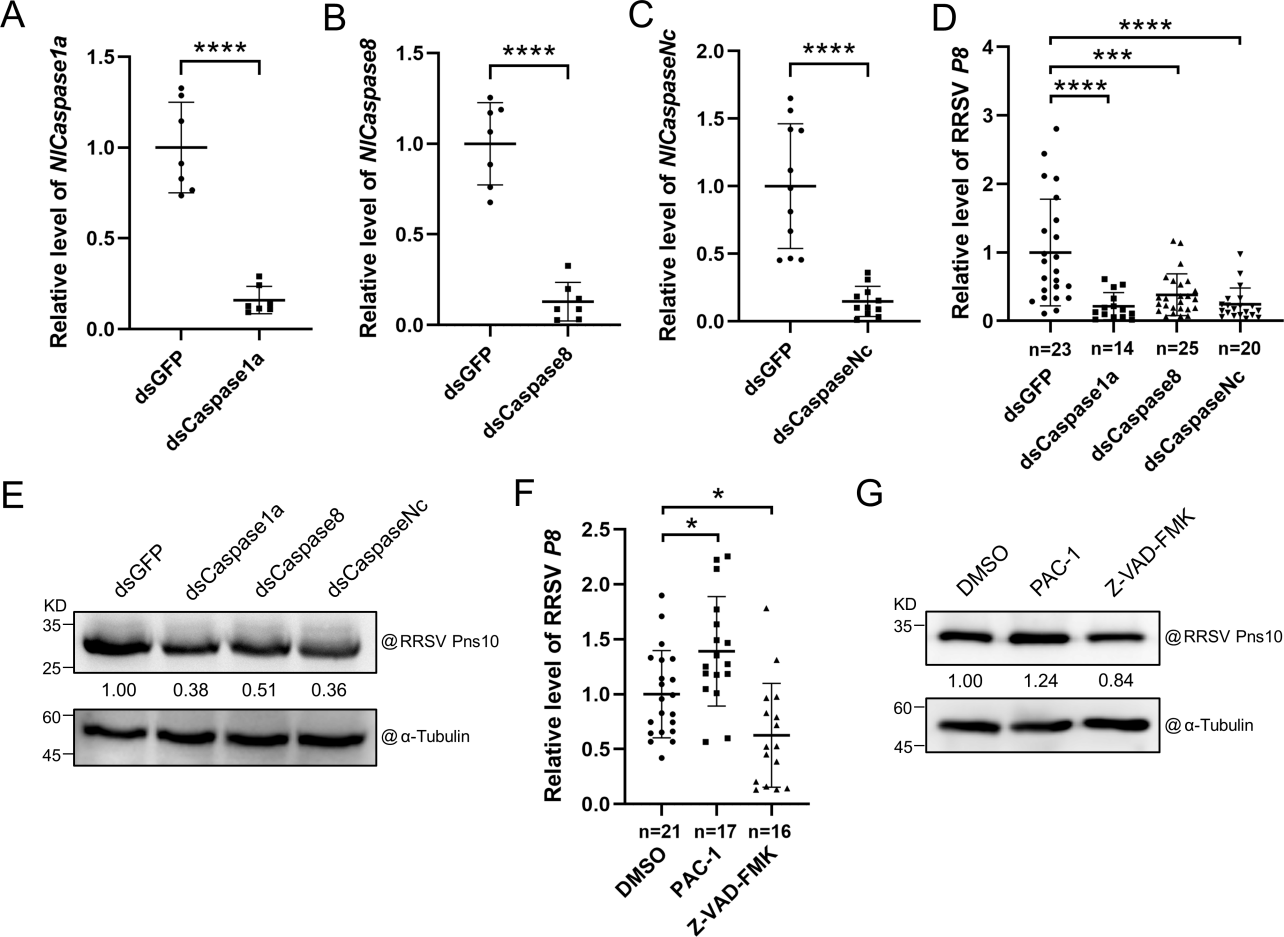
Apoptosis promotes RRSV accumulation in *N. lugens.* **(A-C)** Silencing of *NlCaspase1a*, *NlCaspase8* or *NlCaspaseNc* expression in *N. lugens* was achieved through dsRNA microinjection. Two days later, the expression levels of *Caspase1a*, *Caspase8*, and *CaspaseNc* in microinjected *N. lugens* were determined, respectively, through RT-qPCR. The expression level of *NlActin* was also analyzed and used as the internal control. The values are means ± SDs (A, n = 7; B, n = 7; C, n = 11), determined by the Student’s *t* test. **** indicates *P* < 0.0001. **(D)** RT-qPCR result showing the accumulation level of RRSV RNA in microinjected *N. lugens* at 4 dpvf. The values are the means ± SDs (n = 23; n = 14; n = 25; n = 20), determined by the one-way ANOVA followed by the Tukey’s multiple comparison test. *** indicates **P* *< 0.001 and **** indicates *P* < 0.0001. **(E)** Western blot assay result showing the accumulation level of RRSV Pns10 in microinjected *N. lugens* at 4 dpvf. The α-Tubulin antibody-labeled blot is used to show sample loadings. **(F)** RT-qPCR result showing the accumulation level of RRSV RNA in PAC-1- or Z-VAD-FMK-microinjected *N. lugens* at 4 dpm. The DMSO-microinjected *N. lugens* was used as the control. The values are the means ± SDs (n = 21; n = 17; n = 16), determined by the one-way ANOVA followed by the Tukey’s multiple comparison test. * indicates **P* *<* *0.05. **(G)** Western blot assay result showing the accumulation level of RRSV Pns10 in DMSO-, PAC-1- or Z-VAD-FMK-microinjected *N. lugens* at 4 dpvf. The DMSO-microinjected *N. lugens* were used as the control. The α-Tubulin antibody-labeled blot is used to show sample loadings.

To investigate the effect of apoptosis on RRSV accumulation in *N. lugens*, we synthesized three dsRNA fragments (dsCaspase1a, dsCaspase8, and dsCaspaseNc) representing a partial sequence of *NlCaspase1a*, *NlCaspase8* or *NlCaspaseNc*. The synthesized dsRNA fragments were individually microinjected into *N. lugens*. The *N. lugens* microinjected with ds*GFP* was used as the control. At 2 days post microinjection (dpm), the assayed *N. lugens* were collected and analyzed for the expression levels of *NlCaspase1a*, *NlCaspase8*, and *NlCaspaseNc* through RT-qPCR. The results demonstrated that the expression levels of these three *caspase* genes in dsCaspase1a-, dsCaspase8-, and dsCaspaseNc-microinjected *N. lugens* were significantly silenced when compared to dsGFP-microinjected *N. lugens* (Fig 2A–2C). The dsCaspase1a-, dsCaspase8-, dsCaspaseNc*-* or dsGFP-microinjected *N. lugens* were then allowed to feed on the RRSV-infected rice plants for 4 days followed by RRSV accumulation analysis through RT-qPCR and Western blot assay. The results showed that the accumulation levels of RRSV RNA and Pns10 protein were significantly reduced in dsCaspase1a-, dsCaspase8- or dsCaspaseNc-microinjected *N. lugens* (Fig 2D and 2E), indicating that the silencing of these gene expression inhibits RRSV replication. In a separate experiment, *N. lugens* were microinjected with a procaspase-activating compound-1 (PAC-1) or a caspase inhibitor (Z-VAD-FMK). The DMSO-microinjected *N. lugens* were used as the control. After 24 hours post microinjection, the microinjected *N. lugens* were transferred on RRSV-infected rice plants and allowed to feed for 4 days followed by RRSV accumulation analysis through RT-qPCR and Western blot assay. The results revealed that the accumulation levels of RRSV RNA and Pns10 protein were significantly increased in the PAC-1-microinjected *N. lugens*, but significantly decreased in the Z-VAD-FMK-microinjected *N. lugens* (Fig 2F and 2G), indicating that the RRSV infection-induced apoptotic response in *N. lugens* promotes RRSV accumulation.

### RRSV Pns10 induces apoptosis in Sf9 cells

**Fig 3:**
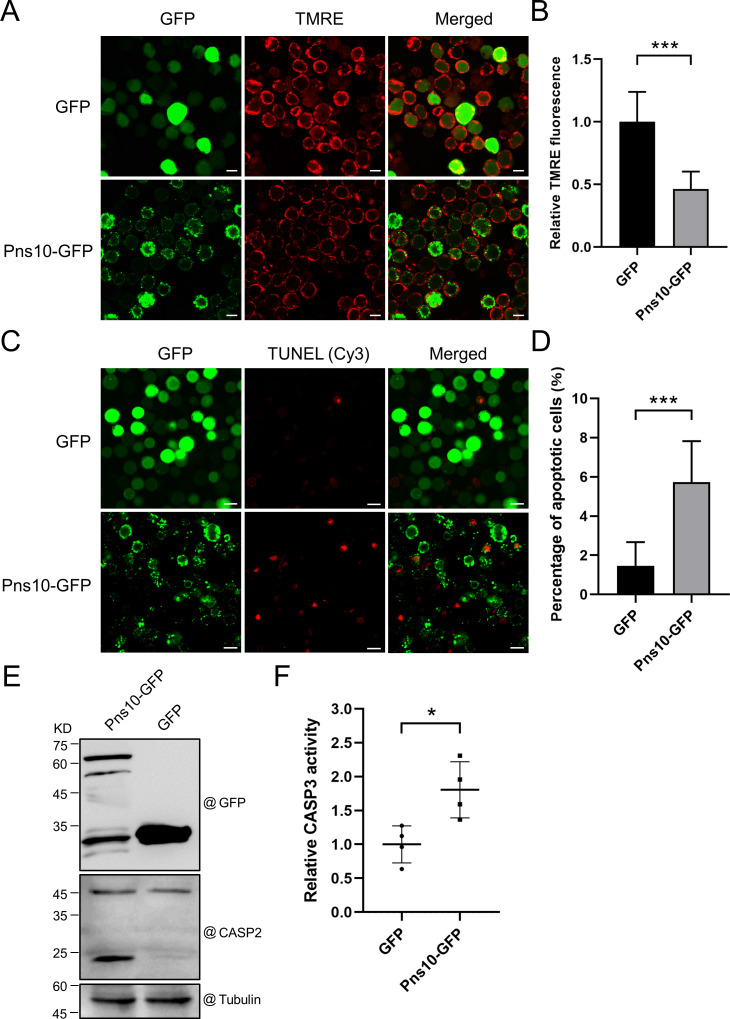
RRSV Pns10 induces apoptosis in Sf9 cells. **(A)** Confocal images showing GFP fluorescence (green) and TMRE fluorescence (red) signals at 3 days post transfection (dpt). Scale bar = 20 μm. **(B)** Analysis of TMRE fluorescence signal intensity using images shown in Fig **A.** The values are the means ± SDs (n = 7), determined by Student’s *t* test. *** indicates **P* *< 0.001. **(C)** Confocal images showing the TUNEL staining signal (Cy3, red) from GFP or Pns10-GFP expressing Sf9 apoptotic cells. Scale bar = 20 μm. **(D)** The percentage of apoptotic cells in GFP or Pns10-GFP expressing Sf9 cell samples. The values are the means ± SDs (n = 8), determined by Student’s *t t*est. ***, **P* *< 0.001. **(E)** Western blot analysis of apoptotic response in Pns10-GFP or GFP expressing Sf9 cells using a CASP2 antibody. A α-tubulin antibody is used to show sample loadings. **(F)** Analysis of CASP3 activity in Pns10-GFP or GFP expressing Sf9 cells using a caspase 3 activity assay kit. The values are the means ± SDs (n = 4), determined by Student’s *t* tes*t*. *, **P* *<* *0.05.

Anti-apoptotic protein BCL2 is a key regulator of mitochondria-mediated apoptosis, and the degradation of BCL2 can trigger mitochondrial apoptosis response [9,28,29]. Because RRSV infection caused the degradation of BCL2 in *N. lugens* (Fig 1C) and RRSV Pns10 has been shown to interact with oligomycin-sensitivity conferral protein in mitochondria of *N. lugens* cells [26], we hypothesized that Pns10 might be responsible for the mitochondrial apoptosis in *N. lugens*. Also, Chen and others reported previously that one of the early stage characteristics of apoptosis is the disruption of mitochondrial membrane potential [8]. Thus, we expressed the Pns10-GFP fusion protein in Sf9 cells using a baculovirus-based expression vector. Analysis of the mitochondrial membrane potential in expressing Sf9 cells through TMRE and confocal microscopy showed that the mitochondrial membrane potential in the Pns10-GFP expressing Sf9 cells was significantly reduced compared with that in the GFP expressing Sf9 cells (Fig 3A and 3B). Furthermore, the TUNEL staining result showed that the percentage of TUNEL-positive cells in the Pns10-GFP expressing Sf9 cells was significantly higher than that in the GFP expressing Sf9 cells (Fig 3C and 3D). In order to identify the core regions responsible for apoptosis induction, we expressed Pns10 1–138-GFP or Pns10 139–297-GFP fusion proteins in Sf9 cells. The TUNEL staining result indicated that both Pns10 1–138-GFP and Pns10 139–297-GFP could induce apoptosis in Sf9 cells (S1A and S1B Fig). Besides, our analysis results showed that the amount of cleaved-caspase2 (CASP2, an apoptosis marker) was significantly increased in Pns10-GFP expressing Sf9 cells (Fig 3E). As expected, the activity of CASP3 in Pns10-GFP expressing Sf9 cells was significantly higher than that in GFP-expressing Sf9 cells (Fig 3F). These above data demonstrate that RRSV Pns10 induces apoptosis in Sf9 cells.

### RRSV Pns10 interacts with NlNDUFS1 in mitochondria of Sf9 cells and *N. lugens* midgut cells

**Fig 4:**
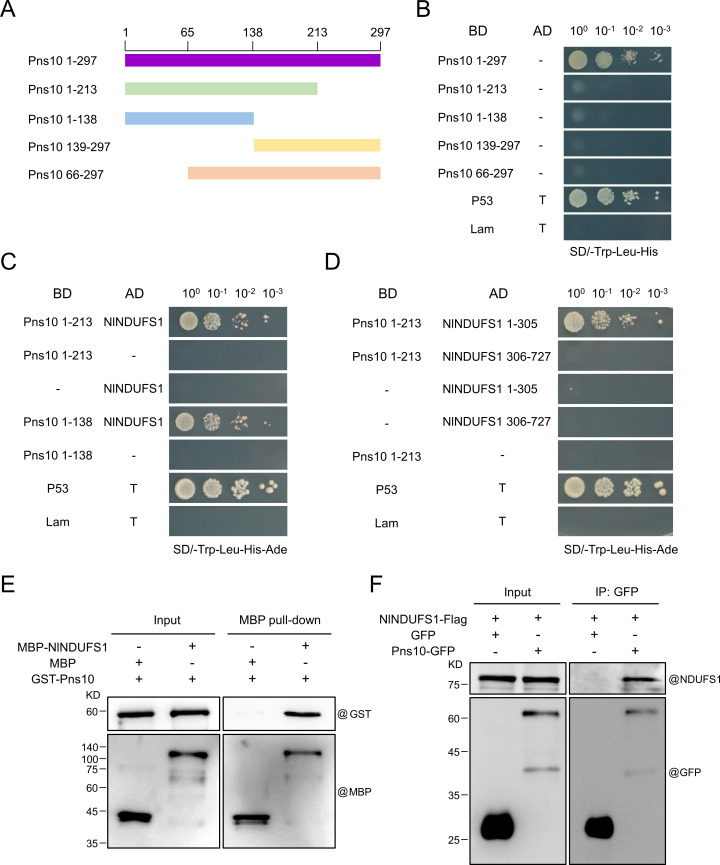
RRSV Pns10 interacts with NlNDUFS1 *in vitro* and *in vivo.* **(A)** Schematic diagrams showing RRSV Pns10 and its deletion mutants. The numbers represent the amino acid (aa) positions in Pns10 and its deletion mutants. **(B)** Self-activation analyses of RRSV Pns10 and its deletion mutants through Y2H assays. Full-length and truncated mutant gene segments of RRSV Pns10 were individually cloned into the pGBKT7 vector and co-transformed with the empty pGADT7 vector into yeast cells. The co-transformed yeast cells were ten-fold diluted and plated on the SD/-Trp-Leu-His medium. The cells co-transformed with pGADT7-T and pGBKT7-p53 or pGADT7-T and pGBKT7-Lam were used as the positive and negative control, respectively. **(C)** Y2H assay result showed that both Pns10 1-213 and Pns10 1-138 interacted with NlNDUFS1. **(D)** Y2H assay result showed that Pns10 1-213 interacted with NlNDUFS1 1-305. **(E)** MBP pull-down assay showing the NlNDUFS1 and Pns10 interaction. Recombinant MBP-NlNDUFS1 or MBP tag protein was incubated with GST-Pns10 and MBP binding beads. Pull-down products were analyzed by Western blot assays using antibodies against GST or MBP. **(F)** Co-IP assay showing the interaction between NlNDUFS1 and Pns10 in Sf9 cells. Sf9 cells co-expressing NlNDUFS1-Flag and GFP or NlNDUFS1-Flag and Pns10-GFP for 3 d, and then whole cell lysates were co-immunoprecipitated with anti-GFP antibody beads. The Co-IP products were analyzed by Western blot assays using anti-NDUFS1 and anti-GFP antibodies.

To explore how RRSV Pns10 triggers apoptosis in *N. lugens*, we first performed yeast two-hybrid (Y2H) assays using RRSV Pns10 as the bait to screen a *N. lugens* cDNA library. Through Y2H, we found that the full-length Pns10 [Pns10 1–297 amino acid (aa)] self-activates in yeast cells, but its truncated mutants, Pns10 1–213 aa, Pns10 1–138 aa, Pns10 139–297 aa and Pns10 66–297 aa cannot (Fig 4A and 4B). Therefore, we used Pns10 1–213 aa as the bait in then subsequent Y2H screens. The Y2H screen results revealed that the Pns10 1–213 aa interacted with a *N. lugens* mitochondrial protein, NADH:ubiquinone oxidoreductase 75 kDa Fe-S protein 1 (NDUFS1). NDUFS1 is a core subunit of mitochondrial complex I, and plays a vital role in cell apoptosis in animals [19,22]. Phylogenetic tree and aa sequence identity analyses using NDUFS1 aa sequences from *N. lugens*, *Laodelphax striatellus*, *Spodoptera frugiperda*, *Homo sapiens*, and *Mus musculus,* retrieved from the NCBI (https://www.ncbi.nlm.nih.gov/) (S1 Table), showed that NDUFS1 is a highly conserved protein across these species, and the *N. lugens* NDUFS1 (NlNDUFS1) shares high aa sequence identity with the other four NDUFS1 proteins, especially the *L. striatellus* NDUFS1 (95.2%) (S2A Fig and S1 Table). Moreover, the Y2H assay results showed that both Pns10 1–213 and Pns10 1–138 interacts with NlNDUFS1 in yeast cells (Fig 4C). Predictions using the UniProt tool (https://www.uniprot.org/) showed that there are three iron-sulfur cluster conserved domains in the N-terminus (aa 1–305) of NlNDUFS1 (S3 Fig). Thus, we investigated the interactions between NlNDUFS1 1–305 or NlNDUFS1 306–727 and Pns10 1–213 through Y2H assays. The results displayed that Pns10 1–213 interacts with NlNDUFS1 1–305 (Fig 4D), but not with NlNDUFS1 306–727. To further confirm this interaction, we performed MBP pull-down and Co-IP assays. The purified recombinant MBP-tagged NlNDUFS1 (MBP-NlNDUFS1) protein was incubated with the purified recombinant GST-tagged RRSV Pns10 (GST-Pns10) or GST protein and MBP beads, the pull-down products were analyzed by Western blot assays using an anti-MBP antibody and anti-GST antibody. The result revealed that GST-Pns10 did interact with MBP-NlNDUFS1, but not with MBP (Fig 4E). Then, we cloned the *Pns10-GFP* and *NlNDUFS1-Flag* genes into the pFastBac dual vector, and simultaneously co-expressed these two proteins in Sf9 insect cells. The cells simultaneously co-expressing GFP and NlNDUFS1-Flag were used as the control. The Sf9 cells co-expressing Pns10-GFP and NlNDUFS1-Flag or GFP and NlNDUFS1-Flag were used for Co-IP assays using anti-GFP magnetic beads. The Co-IP products was analyzed by Western blot assays using anti-NDUFS1 or anti-GFP antibody. The Co-IP result demonstrated that NlNDUFS1-Flag co-immunoprecipitated with Pns10-GFP, but not with GFP (Fig 4F).

**Fig 5:**
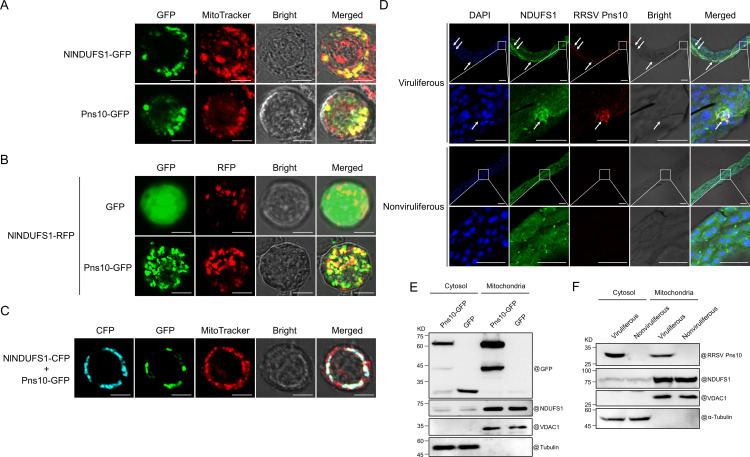
RRSV Pns10 and NlNDUFS1 co-localize in mitochondria of Sf9 cells and *N. lugens* midgut cells. **(A)** Confocal microscopy result showing subcellular localization patterns of NlNDUFS1-GFP and Pns10-GFP in Sf9 cells at 3 dpt. Mitochondria were labeled with MitoTracker (red). Scale bar = 10 μm. **(B)** Subcellular co-localization of Pns10-GFP and NlNDUFS1-RFP or GFP and NlNDUFS1-RFP in Sf9 cells. The cells at 3 dpt were examined and imaged under confocal microscope. Scale bar = 10 μm. **(C)** Co-localization of NlNDUFS1-CFP and Pns10-GFP in mitochondria of Sf9 cells at 3 dpt. Mitochondria were labeled with MitoTracker (red). Scale bar = 10 μm. **(D)** Immunofluorescence assay showing co-localization of RRSV Pns10 and NlNDUFS1 in viruliferous *N. lugens* midgut cells at 4 dpvf. Dylight 549, Dylight 488 and DAPI separately labelled Pns10 (red), NDUFS1 (green) and nuclei (blue). White arrows point to co-localization sites of RRSV Pns10 and NlNDUFS1. Second row is the enlarged images of the boxed areas in upper images. Scale bar = 50 μm. **(E, F)** Analyses of Pns10 and NlNDUFS1 in mitochondria and cytosol fractions from Sf9 cells (E) and *N. lugens* (F) through Western blot assays. In these assays, the voltage-dependent anion channel 1 (VDAC1) was also detected and used as a mitochondrion marker. Tubulin and α-Tubulin were separately detected and used as an Sf9 or *N. lugens* cytosol marker.

To determine subcellular localization patterns of RRSV Pns10 and NlNDUFS1 in mitochondria of Sf9 and *N. lugens* midgut cells, we separately expressed NlNDUFS1-GFP and Pns10-GFP in Sf9 cells and then labeled the mitochondria with MitoTracker (red). Under confocal microscope, both NlNDUFS1-GFP and Pns10-GFP were found to locate in mitochondria of Sf9 cells (Fig 5A). Then, we co-expressed Pns10-GFP and NlNDUFS1-RFP or GFP and NlNDUFS1-RFP (the control) in Sf9 cells. Confocal microscopy result showed that NlNDUFS1-RFP specifically co-localized with Pns10-GFP in mitochondria of Sf9 cells, but not specifically with GFP (Fig 5B). Furthermore, we co-expressed NlNDUFS1-CFP and Pns10-GFP in Sf9 cells using the pFastBac dual vector, and labeled the mitochondria with MitoTracker (red). Confocal microscopy result further confirmed that NlNDUFS1-CFP and Pns10-GFP co-localized in mitochondria of Sf9 cells (Fig 5C). Next, *N. lugens* were allowed to feed on RRSV-infected rice plants for 4 d, and then their midguts were dissected and used to analyze subcellular localizations of RRSV Pns10 and NlNDUFS1 by immunofluorescence assay using a Pns10 monoclonal antibody followed by labeling with a goat anti-mouse IgG conjugated with Dylight 549 and using an NDUFS1 antibody followed by labeling with a goat anti-rabbit IgG conjugated with Dylight 488. The confocal microscopy observation discovered that Pns10 and NlNDUFS1 co-localize in *N. lugens* midgut cells (Fig 5D). Additionally, we performed cytoplasm and mitochondrion isolation from Sf9 cells and *N. lugens*. Western blot assay analyses revealed that RRSV Pns10 is localized in both cytosol and mitochondria fractions of Sf9 cells and *N. lugens* (Fig 5E and 5F), indicating that RRSV Pns10 and NlNDUFS1 are indeed co-localized in mitochondria of Sf9 cells and *N. lugens* cells.

### Silencing *NlNDUFS1* expression induces apoptosis and promotes RRSV infection in *N. lugens*

**Fig 6:**
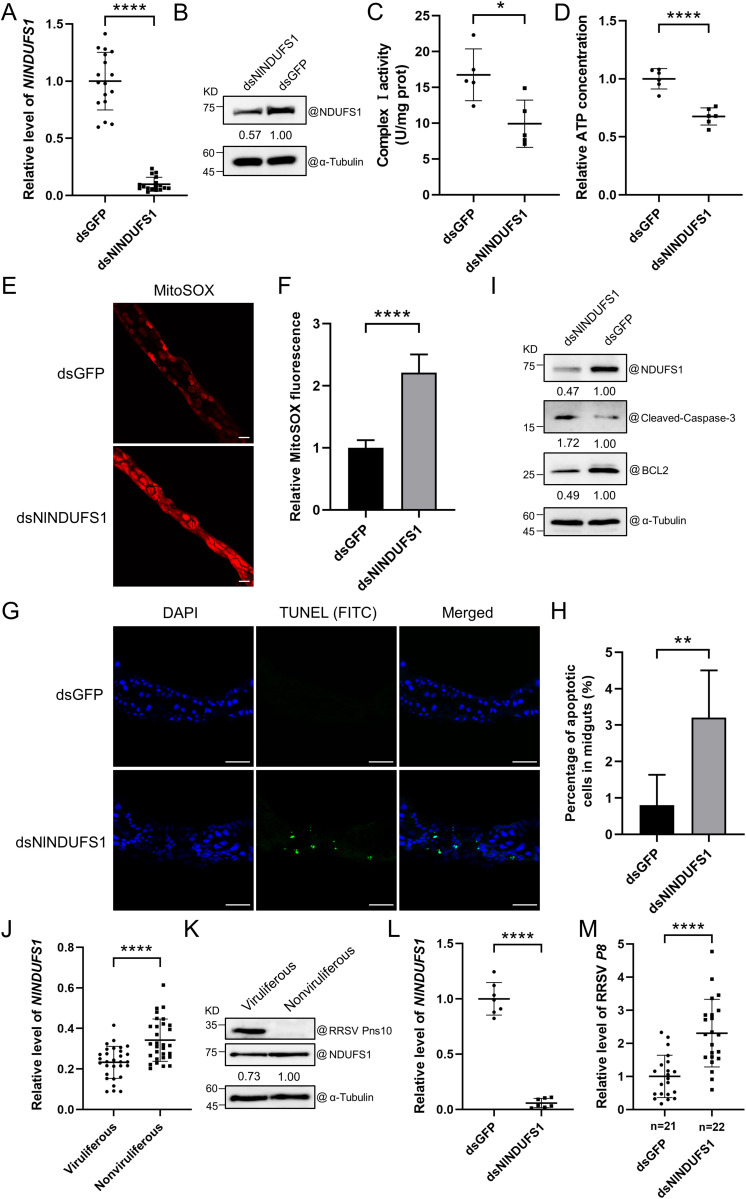
Effect of NlNDUFS1 on apoptosis and RRSV infection in *N. lugens.* **(A, B)** The mRNA (A) and protein (B) levels of *NlNDUFS1* in dsNlNDUFS1- and dsGFP-microinjected *N. lugens* were analyzed by RT-qPCR and Western blot assays at 4 dpm. The expression level of *NlActin* was used as the internal reference. The values are the means ± SDs (n = 17), determined using the Student’s *t* test. **** indicates *P* < 0.0001. α-Tubulin was used as a protein loading control. **(C, D)** Analyses of the complex I activity (C) and ATP production (D) in dsNlNDUFS1- or dsGFP-microinjected *N. lugens* at 4 dpm. The values are means ± SDs (C, n = 5; D, n = 6), determined by Student’s *t t*est. * and **** indicate **P* *<* *0.05 and *P* < 0.0001, respectively. **(E, F)** Analysis of mitochondrial ROS accumulation in the midgut cells of dsNlNDUFS1- or dsGFP-microinjected *N. lugens* at 4 dpm through confocal microscopy. *N. lugens* midguts were stained with MitoSOX (red) and imaged **(E)**. The strength of MitoSOX signal are shown in panel **F.** Scale bar = 50 μm. The values are means ± SDs (n = 7), determined by Student’s *t* test. **** indicates *P* < 0.0001. **(G)** TUNEL staining result showing apoptotic midgut cells in dsNlNDUFS1- or dsGFP-microinjected *N. lugens* at 4 dpm. FITC and DAPI separately labelled TUNEL (green) and nuclei (blue). Scale bar = 50 μm. **(H)** The percentage of apoptotic midgut cells (number of TUNEL-positive cells/number of DAPI-positive cells) in dsNlNDUFS1- or dsGFP-microinjected *N. lugens*. The values are means ± SDs (n = 5), determined by Student’s *t* test. ** indicates **P* *< 0.01. **(I)** Western blot assay results showing expression levels of NlNDUFS1, Cleaved-caspase-3, and BCL2 in dsNlNDUFS1*-* or dsGFP-microinjected *N. lugens* at 6 dpm. α-Tubulin was used as a protein loading control. **(J, K)** The mRNA (J) and protein (K) levels of *NlNDUFS1* in nonviruliferous and viruliferous *N. lugens* determined by RT-qPCR and Western blot assays at 6 dpvf*.* The expression level of *NlActin* was used as an internal control. The values are means ± SDs (n = 30), determined by Student’s *t* test. **** indicates *P* < 0.0001. α-Tubulin was used as a protein loading control. **(L)** RT-qPCR result showing the expression level of *NlNDUFS1* in dsNlNDUFS1- or dsGFP-microinjected *N. lugens* at 2 dpm. The expression level of *NlActin* was used as an internal control. The values are means ± SDs (n = 7), determined by Student’s *t* test. **** indicates *P* < 0.0001. **(M)** RT-qPCR result showing the accumulation of RRSV RNA in dsNlNDUFS1- or dsGFP-microinjected *N. lugens* at 4 dpvf. The values are means ± SDs (n = 21; n = 22), determined by Student’s *t* test. **** indicates *P* < 0.0001.

Several reports have indicated that NDUFS1 plays a crucial role in mitochondrial complex I activity, mitochondrion ATP and ROS production, and induction of cell apoptosis in animal cells [19,22]. To investigate the role of NlNDUFS1 in *N. lugens*, we synthesized dsRNA of *NlNDUFS1* gene partial segment (dsNlNDUFS1) and microinjected it into *N. lugens* to silence NlNDUFS1 expression. A dsGFP was also synthesized and microinjected into *N. lugens* as a control. The RT-qPCR and Western blot assay results showed that compared with dsGFP-microinjected *N. lugens*, the mRNA and protein levels of *NlNDUFS1* in dsNlNDUFS1-microinjected *N. lugens* were significantly reduced at 4 dpm (Fig 6A and 6B). In this experiment, we also detected mitochondrial complex I activity and ATP production in microinjected *N. lugens* at 4 dpm. The result showed that compared with the dsGFP-microinjected *N. lugens*, mitochondrial complex I activity and ATP production in the dsNlNDUFS1-microinjected *N. lugens* were significantly reduced (Fig 6C and 6D). The mitochondrial ROS level in the midgut cells of silenced *N. lugens* at 4 dpm were determined by MitoSOX staining, and confocal microscopy result revealed that the mitochondrial ROS level increased significantly in the midguts of dsNlNDUFS1-microinjected *N. lugens* compared to dsGFP-microinjected controls (Fig 6E and 6F). Furthermore, the TUNEL assay was conducted to detect cell apoptosis in silenced *N. lugens* midgut cells at 4 dpm. Compared with dsGFP-microinjected controls, more positive apoptotic signals (green) were observed in dsNlNDUFS1-microinjected *N. lugens* midgut cells under confocal microscopy (Fig 6G and 6H). Additionally, Western blot assay result showed that the silencing of *NlNDUFS1* expression caused an accumulation of cleaved-caspase-3 and a decrease of BCL2 in dsNlNDUFS1-microinjected *N. lugens* at 6 dpm (Fig 6I). These above findings indicate that the silencing of *NlNDUFS1* expression reduces mitochondrial complex I activity and ATP production, and increases mitochondrial ROS accumulation, thereby inducing apoptosis in *N. lugens*.

To further investigate the effect of RRSV infection on *NlNDUFS1* expression, we performed RT-qPCR and Western blot assays at 6 dpvf. The results displayed that both mRNA and protein levels of *NlNDUFS1* were significantly reduced in viruliferous *N. lugens* compared with nonviruliferous controls (Fig 6J and 6K). In addition, we investigated the role of NlNDUFS1 in RRSV infection. The dsNlNDUFS1-microinjected *N. lugens* were allowed to feed on RRSV-infected rice plants for 4 days, and then analyzed for RRSV RNA accumulation through RT-qPCR using RRSV P8 RNA specific primers. The result showed that at 2 dpm, the mRNA level of *NlNDUFS1* was significantly reduced in dsNlNDUFS1-microinjected *N. lugens* compared to dsGFP-microinjected controls (Fig 6L). The RT-qPCR result also showed that compared with the dsGFP-treated control, the level of RRSV RNA in dsNlNDUFS1*-*microinjected *N. lugens* was significantly increased at 4 dpvf (Fig 6M). These results indicate that the silencing of *NlNDUFS1* expression induces apoptosis and promotes RRSV infection in *N. lugens.*

### RRSV Pns10 reduces mitochondrial complex I activity to suppress ATP production and increase ROS production in Sf9 cells and *N. lugens* cells

**Fig 7:**
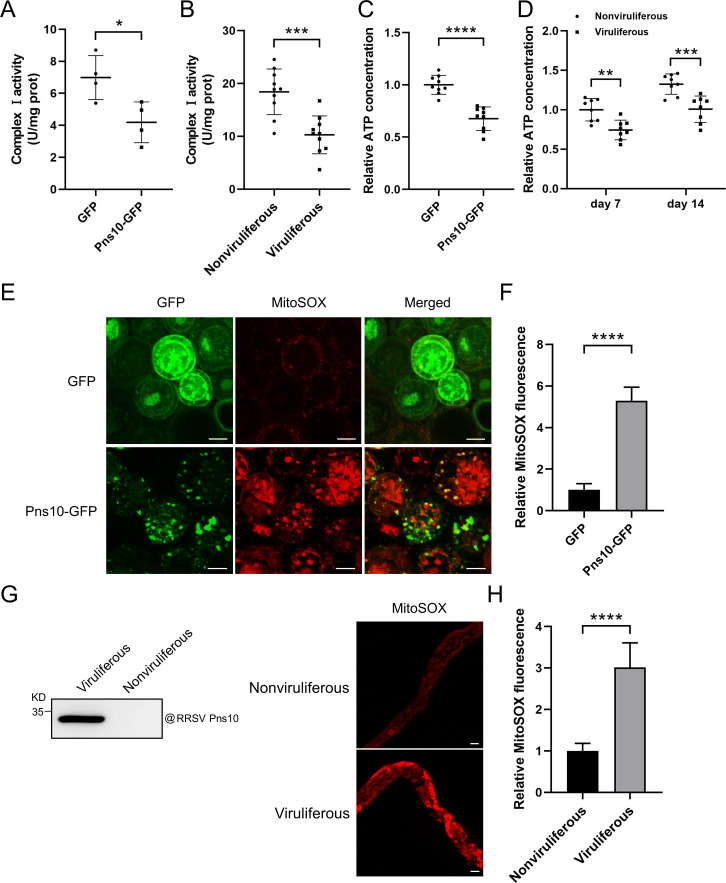
Analyses of the mitochondrial complex I activity and ATP and ROS productions in Pns10-GFP expressing Sf9 cells and RRSV viruliferous *N. lugens.* **(A)** Analysis result showing that the mitochondrial complex I activity in Pns10-GFP expressing Sf9 cells was significantly reduced. The values are means ± SDs (n = 4), determined by Student’s *t* test. * indicates **P* *<* *0.05. **(B)** Analysis result showing the mitochondrial complex I activity in viruliferous *N. lugens* was significantly reduced at 10 dpvf. The values are means ± SDs (n = 10), determined by Student’s *t* test. *** indicates **P* *< 0.001. **(C)** Analysis result showing that ATP production in Pns10-GFP expressing Sf9 cells was significantly reduced. The values are means ± SDs (n = 9), determined by Student’s *t* tes*t*. **** indicates *P* < 0.0001. **(D)** Analysis result showing ATP production in RRSV viruliferous *N. lugens* were significantly reduced at both 7 and 14 dpvf. The values are means ± SDs (n = 8), determined by Student’s *t* test. ** indica*t*es **P* *< 0.01, *** indicates *P* < 0.001. **(E, F)** Confocal microscopy results showing the accumulation level of mitochondrial ROS (MitoSOX, red) in Pns10-GFP or GFP expressing Sf9 cells. Scale bar = 10 μm. The relative strength of MitoSOX florescence signal was also measured through the ImageJ software **(F)**. The values are the means ± SDs (n = 8), determined by Student’s *t* test. **(G, H)** Confocal microscopy results showing the accumulation level of mitochondrial ROS (MitoSOX, red) in midguts of viruliferous and nonviruliferous *N. lugens* at 7 dpvf **(G)**. Western blot assay analyzing RRSV infection in viruliferous and nonviruliferous *N. lugens* using anti-Pns10 monoclonal antibody (left panel in Fig **G)**. Scale bar = 50 μm. The relative strength of MitoSOX florescence signal was measured through the ImageJ software **(H)**. The values are means ± SDs (n = 13), determined by Student’s *t* test.

Because our initial data showed that RRSV Pns10 interacts with NlNDUFS1, and silencing of *NlNDUFS1* expression reduces mitochondrial complex I activity and ATP production, but increases mitochondrial ROS accumulation in *N. lugens* (Figs 4 and 6), we hypothesized that RRSV Pns10 may reduce mitochondrial complex I activity through interacting with NlNDUFS1 to decrease ATP production and to increase ROS generation in *N. lugens*. To test this hypothesis, we expressed Pns10-GFP or GFP in Sf9 cells, and then analyzed mitochondrial complex I activity in the expressing Sf9 cells. The result demonstrated that the mitochondrial complex I activity in Pns10-GFP expressing Sf9 cells was significantly lower than that in GFP expressing Sf9 cells (Fig 7A), indicating that RRSV Pns10 expression can reduce the mitochondrial complex I activity in Sf9 cells. Then, we further analyzed the mitochondrial complex I activity in RRSV viruliferous and nonviruliferous *N. lugens* at 10 dpvf and found that the mitochondrial complex I activity in viruliferous *N. lugens* was significantly lower than that in nonviruliferous controls (Fig 7B), which suggests that RRSV Pns10 also can reduce the mitochondrial complex I activity in *N. lugens.* Additionally, we analyzed ATP production in Pns10-GFP or GFP expressing Sf9 cells and found that ATP production was significantly reduced in Pns10-GFP expressing Sf9 cells compared to GFP expressing controls (Fig 7C). To further validate this finding, we analyzed ATP production in viruliferous and nonviruliferous *N. lugens* at 7 and 14 dpvf. The results showed that after RRSV infection, ATP production in *N. lugens* were significantly reduced at both 7 and 14 dpvf (Fig 7D). The MitoSOX staining and confocal microscopy result showed that the mitochondrial ROS accumulation was significantly increased in Pns10-GFP expressing Sf9 cells compared to GFP expressing controls (Fig 7E and 7F). We also tested ROS accumulation in viruliferous and nonviruliferous midgut cells of *N. lugens* at 7 dpvf, and the result showed that RRSV infection increased ROS accumulation in *N. lugens* midgut cells (Fig 7G and 7H). Taken together, these our findings indicate that RRSV infection and Pns10 expression can reduce ATP production and increase ROS generation in Sf9 cells and *N. lugens* midgut cells through the Pns10-NlNDUFS1 interaction to reduce mitochondrial complex I activity.

### RRSV Pns10 interferes the interaction between NlNDUFS1 and NlPHB2

**Fig 8:**
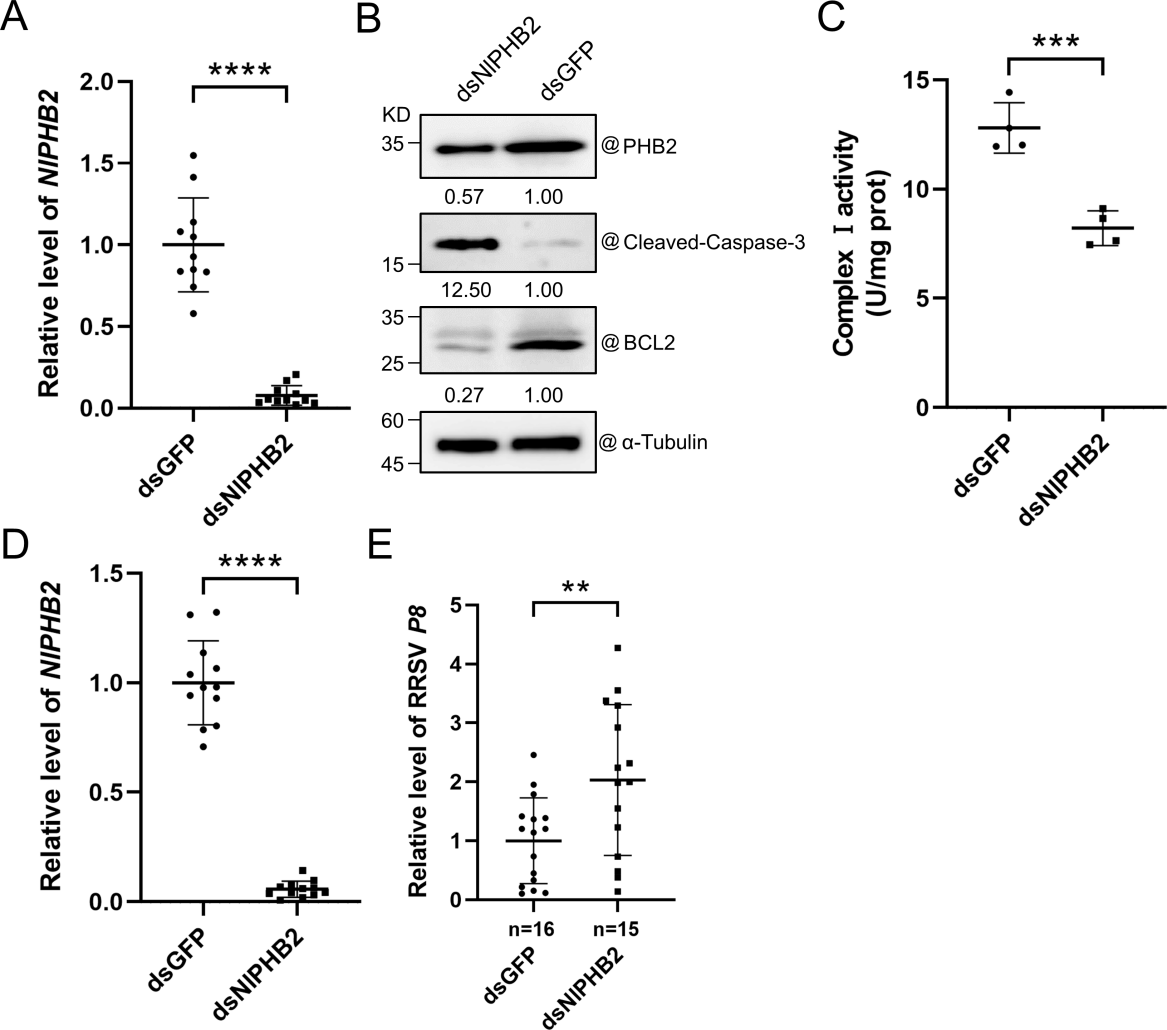
Effect of NlPHB2 on apoptosis and RRSV infection in *N. lugens.* **(A)** Result of RT-qPCR showing the expression level of *NlPHB2* in dsNlPHB2- or dsGFP-microinjected *N. lugens* at 4 dpm. The expression level of *NlActin* was used as an internal reference. The values are the means ± SDs (n = 11), determined by Student’s *t* test. **** indicates *P* < 0.0001. **(B)** Results of Western blot assays showing the accumulations of NlPHB2, Cleaved-caspase-3, and BCL2 in dsNlPHB2*-* or dsGFP-microinjected *N. lugens* at 4 dpm. α-Tubulin is used to show sample loadings. **(C)** Analysis of complex I activity in dsNlPHB2*-* or dsGFP-microinjected *N. lugens* at 4 dpm. The values are means ± SDs (n = 4), determined by Student’s *t* test. *** indicates **P* *< 0.001. **(D)** Result of RT-qPCR showing the expression level of *NlPHB2* in dsNlPHB2- or dsGFP-microinjected *N. lugens* at 2 dpm. The expression level of *NlActin* was used as an internal reference. The values are means ± SDs (n = 12), determined by Student’s *t* tes*t*. **** indicates *P* < 0.0001. **(E)** Result of RT-qPCR showing the accumulation level of RRSV RNA in dsNlPHB2- or dsGFP-microinjected *N. lugens* at 4 dpvf. The expression level of *NlActin* was used as an internal reference. Sample sizes (n) for statistical tests indicated in panels refer to biologically independent *N. lugens*. The values are means ± SDs (n = 16, 15), determined by Student’s *t* test. ** indica*t*es **P* *< 0.01.

A previous report revealed that the loss of Prohibitin 2 (PHB2) function causes mitochondria fragmentation and disordering, and induces apoptosis [30]. In this study, we investigated the effect of NlPHB2 on mitochondrial complex I activity and apoptosis in *N. lugens*. We first analyzed phylogenetic relationships and aa sequence identity between NlPHB2 (XM_039420816.1) and other four species PHB2 orthologs from NCBI (S2 Table). The results showed that PHB2 is a highly conserved protein across these species, and NlPHB2 shares high aa sequence identity with the other four PHB2 orthologs (S2B Fig and S2 Table). Subsequently, we cloned *NlPHB2* gene from the total RNA extracted from *N. lugens* through RT-PCR, and then produced dsRNA of*NlPHB2* gene partial fragment (dsNlPHB2). The dsNlPHB2 was microinjected into *N. lugens* to silence the expression of *NlPHB2*. *N. lugens* microinjected with dsGFP were used as the control. Results of RT-qPCR and Western blot assay showed that compared with dsGFP-microinjected controls, mRNA and protein levels of *NlPHB2* were significantly reduced in dsNlPHB2-microinjected *N. lugens* at 4 dpm (Fig 8A and 8B). We then analyzed the mitochondrial complex I activity and found that the mitochondrial complex I activity in dsNlPHB2-microinjected *N. lugens* was significantly reduced at 4 dpm (Fig 8C). To investigate the role of *NlPHB2* in apoptosis, we performed Western blot assays. The results showed that the silencing of *NlPHB2* expression in *N. lugens* caused an accumulation of cleaved-caspase-3 and a degradation of BCL2 at 4 dpm (Fig 8B). These above findings indicate that silencing *NlPHB2* expression reduced the mitochondrial complex I activity and induced apoptosis in *N. lugens*.

To further investigate the effect of RRSV infection on NlPHB2 expression, we performed RT-qPCR and Western blot assays at 6 dpvf. The results displayed that compared to nonviruliferous controls, viruliferous *N. lugens* exhibited comparable NlPHB2 expression at both mRNA and protein levels (S4A and S4B Fig). Furthermore, we investigated the role of NlPHB2 on RRSV infection. The dsNlPHB2- or dsGFP-microinjected *N. lugens* were allowed to feed on the RRSV-infected rice plants for 4 days followed by RT-qPCR. The result displayed that the expression of *NlPHB2* in dsNlPHB2-microinjected *N. lugens* was significantly downregulated at 2 dpm (Fig 8D), whereas the accumulation of RRSV RNA was significantly increased at 4 dpvf (Fig 8E). These findings indicate that the silencing of *NlPHB2* expression promotes RRSV replication in *N. lugens.*

**Fig 9:**
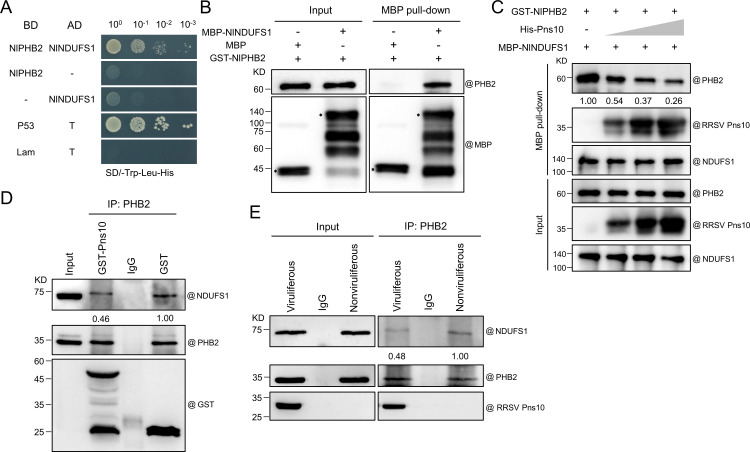
RRSV Pns10 disrupts the NlNDUFS1 and NlPHB2 interaction in *N. lugens.* **(A)** A Y2H assay result showing the interaction between NlPHB2 and NlNDUFS1. Full-length *NlPHB2* and *NlNDUFS1* gene sequences were separately cloned into the pGBKT7 and pGADT7 vectors. After co-transformation into yeast cells, the cells were ten-fold diluted and plated on the SD/-Trp-Leu-His medium. The cells co-transformed with pGADT7-T and pGBKT7-p53 or pGADT7-T and pGBKT7-Lam were used as the positive and negative control. **(B)** MBP pull-down assay result showing the NlNDUFS1 and NlPHB2 interaction. Purified prokaryotic expressed MBP-NlNDUFS1 or MBP tag was incubated with purified prokaryotic expressed GST-NlPHB2, and then with the MBP binding beads. The pull-down products were then analyzed through Western blot assays using a PHB2 or an MBP antibody. **(C)** Competitive MBP pull-down assays showing that RRSV Pns10 interferes with the NlNDUFS1 and NlPHB2 interaction *in vitro*. MBP-NlNDUFS1, His-Pns10, and GST-NlPHB2 were individually expressed in *E. coli*. After purification, different amounts of His-Pns10 were added to the MBP-NlNDUFS1 and GST-NlPHB2 mixed samples. Western blot assays were used to analyze the input and pull-down proteins with an anti-PHB2, anti-RRSV Pns10, or anti-NDUFS1 antibody, and the results showed that RRSV Pns10 disrupted the *in vitro* interaction between NlNDUFS1 and NlPHB2. **(D)** Semi-*in vitro* competitive binding Co-IP assay showing that RRSV Pns10 interferes with the NlNDUFS1 and NlPHB2 interaction in *N. lugens*. Purified prokaryotic expressed GST-Pns10 or GST was co-incubated with *N. lugens* total protein extracts, and then with an anti-PHB2 antibody followed by the rProtein G agarose resins. Western blot assays were used to analyze the input and Co-IP proteins with an antibody against NlNDUFS1, NlPHB2 or GST, and the results showed that the Pns10 disrupts the NlNDUFS1 and NlPHB2 interaction in *N. lugens*. **(E)** *In vivo* competitive binding Co-IP assay showing that RRSV infection interferes with the NlNDUFS1 and NlPHB2 interaction in *N. lugens*. The total proteins extracted from viruliferous or nonviruliferous *N. lugens* were incubated with an anti-PHB2 antibody followed by the rProtein G agarose resins. Western blot assays were used to analyze the input and Co-IP proteins with an antibody against NlNDUFS1, NlPHB2 or Pns10, and the results showed that RRSV infection disrupted the NlNDUFS1 and NlPHB2 interaction in *N. lugens*.

It is known that PHB2 can interact with NDUFS1 to stabilize the mitochondrial complex I and to enhance its activity in human colorectal cancer cells [30]. Thus, we asked whether RRSV Pns10 could disrupt the interaction between NlNDUFS1 and NlPHB2 to affect the mitochondrial complex I activity and to induce apoptosis in *N. lugens*. Y2H and MBP pull-down assays revealed a physical interaction between NlNDUFS1 and NlPHB2 (Fig 9A and 9B)*.* However, the Y2H assay results showed that RRSV Pns10 1–213 did not interact with NlPHB2 (S5 Fig). Through *in vitro* competitive binding pull-down assay, we also found that RRSV Pns10 disrupted the NlNDUFS1 and NlPHB2 interaction in a dosage-dependent manner (Fig 9C). To further confirm this finding, we performed a semi-*in vitro* competitive binding Co-IP assay. Purified prokaryotic expressed Pns10-GST or GST was added to *N. lugens* total protein extracts, and the mixtures were then co-incubated with a PHB2 antibody followed by the rProtein G agarose resins. Analyses of the Co-IP products through Western blot assays showed that Pns10-GST, but not GST alone, disrupted the NlPHB2 and NlNDUFS1 interaction (Fig 9D). Furthermore, we performed a *in vivo* competitive binding Co-IP assay. *N. lugens* were allowed to feed on RRSV-infected rice plants for 10 days, and then their total protein was extracted. The total protein extracted from nonviruliferous *N. lugens* was used as the control. The total protein extracts were then co-incubated with a PHB2 antibody followed by rProtein G agarose resins. Analyses of the Co-IP products through Western blot assays showed that the NlPHB2 and NlNDUFS1 interaction was indeed disrupted in RRSV-infected *N. lugens* (Fig 9E). Collectively, these data indicate that RRSV Pns10 interferes with the interaction between NlNDUFS1 and NlPHB2 in *N. lugens*.

## Discussion

**Fig 10:**
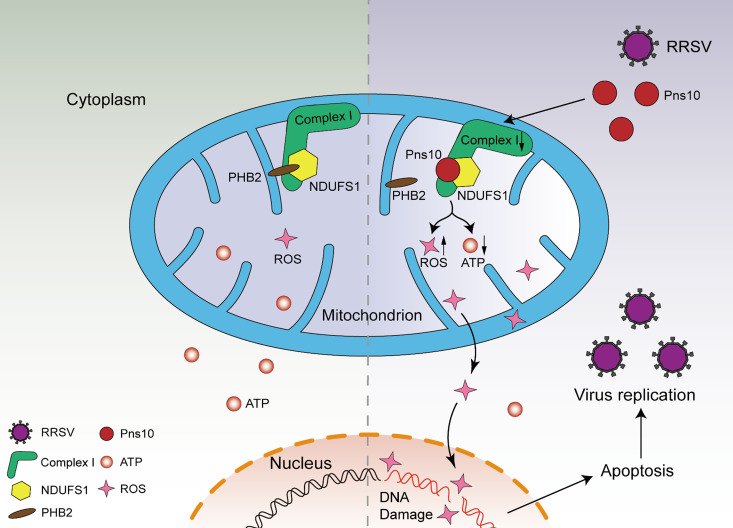
A working model for the RRSV Pns10-promoted RRSV infection in *N. lugens* through inducing NlNDUFS1-mediated mitochondrial apoptosis. RRSV infection activates NlNDUFS1-mediated mitochondrial dysfunction and apoptosis in *N. lugens*. During viral infection, RRSV Pns10 impairs the mitochondrial complex I activity through disrupting the NlNDUFS1 and NlPHB2 interaction. This impairment of mitochondrial complex I activity results in a significant decrease of ATP production and an increase of mitochondrial ROS production, which causes mitochondrial apoptosis to facilitate RRSV infection in *N. lugens*.

Mitochondria-mediated apoptosis is an important pathway for viruses to induce host cell apoptosis [31]. Through this study, we have found that RRSV infection or RRSV Pns10 expression can induce NlNDUFS1-mediated mitochondrial dysfunction and apoptosis to promote virus infection in *N. lugens*. RRSV Pns10 directly interacts with NlNDUFS1 to disrupt the interaction between NlNDUFS1 and NlPHB2 (Figs 4 and 9). This disruption reduces the activity of mitochondrial complex I and reduces ATP production, resulting in an increased mitochondrial ROS accumulation (Fig 7). It was reported that the increased mitochondrial ROS production promotes DNA damage, thereby triggering the mitochondria-mediated apoptosis in animal cells [19,32,33]. In this study, we have also found that the increased mitochondrial ROS production activates apoptosis in *N. lugens*, leading to an enhanced RRSV accumulation in *N. lugens* (Fig 10).

NDUFS1 is a core subunit of mitochondrial complex I and plays an important role in the maintenance of mitochondrial complex I structure and function [17,19]. NDUFS1 contains three conserved iron-sulfur clusters in its N-terminus (aa 1–305) essential for efficient transfer of electrons within mitochondrial complex I [16,17]. In this study, we have found that the Pns10 N-terminus (1–213aa) interacts with the NlNDUFS1 N-terminus (1–305aa) in H2Y assay (Fig 4D). We speculate that the Pns10 binds to the iron-sulfur clusters to interfere the electron transfer of NlNDUFS1 within mitochondrial complex I, leading to the disruption of mitochondrial complex I activity and mitochondrion function. Accumulating evidences have indicated that NDUFS1 is closely associated with cell apoptosis. For example, Ricci and others have reported that NDUFS1 acts as a critical caspase substrate, and the expression of a non-cleavable NDUFS1 mutant in Hela cells can sustain mitochondrial transmembrane potential and ATP levels, and inhibit ROS production in response to apoptotic stimuli [20]. In 2020, Qi and others reported that deficiency of A-kinase anchoring protein 121 (Akap1) in diabetic cardiomyopathy reduces mitochondrial complex I activity through inhibiting the translocation of NDUFS1 from cytosol to mitochondria, which decreases ATP production and increases mitochondrial ROS-mediated apoptosis [34]. More recently, overexpression of NDUFS1 alleviates myocardial infarction, hypoxia-induced ROS production, and ROS-related apoptosis [22]. Mouse double minute 2 (MDM2) has also been shown to interact with NDUFS1 to prevent its mitochondrial localization, resulting in destabilization of mitochondrial complex I and supercomplex, the increase of ROS production, and the BIM-mediated BAK/BAX-dependent activation of the mitochondrial apoptosis [19]. Here, we provide evidences to show that RRSV manipulates the NDUFS1-mediated mitochondrial apoptosis to promote its infection in *N. lugens*.

Previous studies have shown that RRSV Pns10 is a component of viroplasm and has ATPase activity [24,26], and the point mutation (K20Q) in Pns10 abolishes its ATPase activity [35]. To explore whether this enzymatic function directly contributes to mitochondrial energy disruption or ROS accumulation, we constructed two Pns10^K20Q^ 1–213 and Pns10^K20Q^ 1–138 mutants, and verified their interaction with NlNDUFS1. The Y2H assay result showed that both Pns10^K20Q^ 1–213 and Pns10^K20Q^ 1–138 still interact with NlNDUFS1 (S6A Fig), and also disrupt mitochondrial ATP production (S6B Fig) and increase ROS accumulation in Sf9 cells (S6C and S6D Fig). These results demonstrate that ATPase activity of Pns10 does not contribute to mitochondrial energy disruption or ROS accumulation. Beyond RRSV Pns10, non-structural proteins encoded by some plant reovirus are also known to have ATPase activity. For example, the Pns6 of rice dwarf virus (RDV) and the Pns7 of rice gall dwarf virus (RGDV), two members in the genus *Phytoreovirus*, as well as the P9-1 of mal de Río Cuarto virus (MRCV), a member in the genus *Fijivirus*, possess ATPase activity [36–39]. These non-structural proteins with ATPase activity are components of viroplasm or movement proteins, which seems to indicate that these non-structural proteins can manipulate host to provide energy for virus replication or movement. It is well known that mitochondria are the main sites to provide energy for various life activities. A previous report by Huang and others showed that RRSV Pns10 interacts with a mitochondrial protein oligomycin-sensitivity conferral protein (OSCP), a component of the mitochondrial F_0_F_1_ proton ATP synthase/ATPase (F-type H + -ATPase), to promote RRSV infection in *N. lugens* [26]. In this study, we have found that RRSV Pns10 can also interact with NlNDUFS1, a mitochondrial complex I protein, to reduce mitochondrial complex I activity and ATP synthesis (Fig 8). Collectively, RRSV Pns10 with ATPase activity can interplay directly with mitochondrial energy metabolism-associated proteins to involve in host energy metabolism.

Nowadays, the molecular mechanism by which plant arboviruses mediate apoptosis in host cells has rarely been reported. Chen et al. discovered that RGDV Pns11-induced fibrillar structure could target mitochondria and induce apoptotic response to facilitate RGDV infection in its vector leafhopper (*Recilia dorsalis*) [8]. The Pns11 interacts with the mitochondrial apoptosis-related protein VDAC1 and targets to the outer membrane of mitochondria, causing mitochondrial degeneration and membrane potential decrease, thereby leading to cell apoptosis [8]. The interaction of Pns11-VDAC1-GSN induces mitochondrial-mediated cell apoptosis by controlling the release of apoptotic signaling molecules such as cytochrome c through VDAC1 porin [40]. Rice stripe virus (RSV) and rice black-streaked dwarf virus (RBSDV) infection activate JAK-STAT signaling pathway in *Laodelphax striatellus* to induce the mitochondria-mediated apoptosis, which enhances their replications [9]. The SOCS5 (suppressor of cytokine signaling 5), an E3 ubiquitin ligase regulated by STAT5B (signal transducer and activator of transcription 5B), directly interacts with anti-apoptotic BCL2 and accelerates BCL2 degradation through the 26S proteasome pathway [9]. This process allows the release of cytochrome c from mitochondria into cytosol and induces subsequent apoptosis, resulting in the enhancement of viral replication [9]. Through this study, we have found that RRSV Pns10 can target mitochondria and interact with NlNDUFS1, a core subunit of mitochondrial complex I, to disrupt mitochondrial function, thereby leading to mitochondrial apoptosis and promoting RRSV infection in *N. lugens* (Fig 10). Thus, both previous reports and our researches indicate that plant arboviruses can directly or indirectly target host cell mitochondria and disrupt their normal function, thereby inducing mitochondrial apoptosis and promoting viral infection. However, to date, how apoptosis promotes viral infections in their insect vectors are mostly unknown. Previous studies have found that African swine fever virus (ASFV) and avian influenza virus (AIV) can hijack the apoptotic uptake pathway and use apoptotic bodies for virus spread between cells and for immune escape [41,42]. In this work, we have discovered that RRSV can manipulate the apoptotic response to benefit its infection in its insect vector (Figs 1 and 2). However, whether RRSV and other persistently transmitted plant arboviruses can also utilize apoptotic bodies to spread and to escape insect immune responses remains to be determined.

When *N. lugens* feeds on RRSV-infected rice plants, RRSV enters epithelial cells of *N. lugens* midguts through esophaguses and moves sequentially to hemolymph, and salivary glands [24,43]. After this circulation period, RRSV is secreted along with saliva into uninfected rice plants to achieve horizontal transmission. Huang and colleagues found that the silencing of *NlCaspase1* genes caused a significant reduction of RRSV transmission efficiency from insect vector to rice plants [27]. Collectively, RRSV-induced apoptosis not only promotes viral infection in *N. lugens*, but also facilitates viral horizontal transmission from insect vectors to rice plants. This strategy enables RRSV to establish a stable transmission cycle in the *N. lugens*-rice ecosystem. The development of RRSV mutants remains technically challenging due to the current lack of established reverse genetics systems for plant reoviruses. Future studies should focus on developing a reverse genetics system for RRSV to elucidate its molecular interactions with *N. lugens* and its pathogenicity.

## Materials and methods

### Virus source and rice inoculation and growth conditions

Rice plants showing RRSV symptoms were collected from paddy fields in Hainan Province of China. RRSV infection in these collected plants was determined through RT-PCR with RRSV-specific primers. The confirmed RRSV-infected rice plants were maintained in a greenhouse set at 28–32°C and a 16 h light/8 h dark photoperiod. To produce new RRSV-infected rice plants for further studies, second-instar *N. lugens* nymphs were allowed to feed on RRSV-infected rice plants for 3 days and then on healthy rice seedlings for 10 days. The inoculated rice seedlings were grown inside the greenhouse for 15 days and then tested for RRSV infection through RT-PCR. Nonviroliferous *N. lugens* were maintained on healthy rice seedlings grown inside an insect culture room maintained at 25°C and a 16 h light/8 h dark photoperiod.

### Protein extraction and Western blot assay

For western blot assays, *N. lugens* protein was extracted using a RIPA lysis buffer (Beyotime, Shanghai, China) and Sf9 cell protein was extracted using a Cell lysis buffer for Western and IP (Beyotime). The resulting protein samples were mixed with a 5 × SDS loading buffer (Fdbio science, Hangzhou, China), boiled for 10 min, and then separated in SDS-PAGE gels. A RRSV Pns10 monoclonal antibody was generated in our laboratory. The Cleaved-Caspase-3 antibody was purchased from the Cell Signaling Technology (Danvers, MA, USA), and the BCL2 and GST antibodies were purchased from the Huabio (Hangzhou, China). The α-tubulin antibody was purchased from the Beyotime (Shanghai, China). The MBP, NDUFS1, and PHB2 antibodies were purchased from the ABclonal (Wuhan, China). The GFP antibody was purchased from the Abcam (Cambridge, UK) and the horse radish peroxidase (HRP)-conjugated second antibody was purchased the Sigma-Aldrich (Taufkirchen, Germany). After protein transfer to nitrocellulose membranes, the membranes were probed with a specific primary antibody solution diluted at 1:5,000 (v/v) for 1 h at 37°C. After three times washes in a 0.01 M phosphate-buffered saline (PBS, 140 mM NaCl, 2.7 mM KCl, 10 mM KH_2_PO_4_, 10 mM Na_2_HPO_4_, 1.8 mM KH_2_PO_4_, pH 7.4), the membranes were probed with the HRP-conjugated second antibody solution diluted at 1:8,000 (v/v) for 1 h at 37°C. After three times washes in a 0.01 M PBS, the detection signal was then visualized using an ECL substrate and captured using an ImageQuant LAS 4000mini instrument (GE HealthCare, Illinois, USA).

### Immunofluorescence microscopy

*N. lugens* midguts were collected and fixed for 2 h in an Immunol staining fix solution (Beyotime) at room temperature (RT). The fixed samples were permeabilized in an Immunostaining permeabilization solution with Triton X-100 (Beyotime) for 1 h. The samples were then incubated in a 1% bovine serum albumin solution for 1 h at RT and then probed overnight with a RRSV Pns10 monoclonal antibody and a NDUFS1 rabbit polyclonal antibody at 4°C. After three times washes in 0.01 M PBS, the samples were incubated in a solution containing a goat anti-mouse IgG conjugated with Dylight 549 (goat anti-mouse IgG-Dylight 549, EarthOx, San Francisco, CA, USA) and a goat anti-rabbit IgG conjugated with Dylight 488 (goat anti-rabbit IgG-Dylight 488, EarthOx) for 2 h at RT. Nuclei in midgut cells were stained with Antifade mounting medium with DAPI (Beyotime) as instructed. The treated samples were examined and imaged under a FV3000 Olympus confocal microscope (Olympus, Tokyo, Japan).

### Productions of double-stranded RNAs (dsRNAs) and injection

To produce dsRNAs, primers with a T7 RNA polymerase promoter sequence (TAATACGACTCACTATAGGG) at their 5′ termini was designed (S3 Table). DNA fragment representing the partial sequence of *NlNDUFS1* (544 bp, nt position 817–1360), *NlPHB2* (557 bp, nt position 128–684), *NlCaspase1a* (501 bp, nt position 561–1061), *NlCaspase8* (503 bp, nt position 1371–1873) or *NlCaspaseNc* (547 bp, nt position 1121–1667) was individually PCR-amplified from a *N. lugens* cDNA. A 502 bp *GFP* fragment was also amplified. The resulting PCR products were used for *in vitro* transcription using the T7 RNAi Transcription Kit (Vazyme, Nanjing, China) to produce dsRNAs. The quality and size of individual dsRNA product were checked in 1% agarose gels (BioFroxx, Einhausen, Germany) through electrophoresis. For RNAi assay, third-instar *N. lugens* nymphs were injected with 50 nL of the dsRNA product into hemolymph in the insect ventral thorax via a glass needle using a Nanoject Ⅲ instrument (Drummond, Pennsylvania, USA).

### Analyses of gene expression and viral RNA accumulation through RT-qPCR

Total RNA was isolated from assayed *N. lugens* using TRI-Reagent (Invitrogen, Massachusetts, USA). After removal of genomic DNA, the RNA samples (1 μg per 20 μL reaction) were individually reverse-transcribed using the HiScript II Q Select RT SuperMix (Vazyme). The resulting cDNA samples were then used for quantitative PCR (qPCR) using the ChamQ SYBR Color qPCR Master Mix (Vazyme) on a LightCycler 480 system (Roche, Basel, Switzerland). At least three independent RT-qPCR assays were performed for each experiment. The expression of *N. lugens Actin* was used as the internal control. The primers used in this study are listed in S3 Table.

### Apoptosis inhibition and induction assays

Apoptosis inhibitor Z-VAD-FMK (MedChemExpress, New Jersey, USA) and inducer PAC-1 (Selleck Chemicals, Houston, TX, USA) were used to examine the effects of apoptotic pathways on RRSV infection in *N. lugens*. Third-instar *N. lugens* nymphs were first injected with 60 nL of 0.2 mM Z-VAD-FMK, 0.2 mM PAC-1 or 2% Dimethyl sulfoxide (DMSO, control), respectively, and then allowed to feed on healthy rice seedlings for 1 d. These insects were then transferred onto RRSV-infected rice plants to feed for 4 d, and analyzed for viral RNA and protein accumulations through RT-qPCR and Western blot assays, respectively.

### Baculovirus expression assay

Full-length RRSV *Pns10* and **N. lugens* NDUFS1* (*NlNDUFS1*) sequences were RT-PCR-amplified from total RNA of viruliferous *N. lugens*. The resulting DNA products were purified and cloned into the pFastBac or pFastBac dual vector (Invitrogen, Carlsbad, CA, USA). The recombinant baculovirus vectors were transformed individually into *E. coli* DH10Bac cells (Invitrogen, Carlsbad, CA, USA) to produce recombinant bacmid vectors. Sf9 cells were transfected with individual recombinant bacmid vector using LipoInsect Transfection Reagent as instructed (Beyotime). The transfected cells were incubated inside a 27°C humidified incubator for 3 days. Expressions of recombinant proteins in the transfected cells were analyzed through confocal microscopy and Western blot assays.

### Yeast two-hybrid assay (Y2H)

Y2H assays were performed as described previously [44]. Briefly, the target genes were cloned individually into the pGBKT7 or pGADT7 vector. The resulting vectors were co-transformed, in different combinations, into *Saccharomyces cerevisiae* Gold strain cells. The transformed cells were screened on the selective SD double-dropout (DDO) medium (SD/-Leu/-Trp) at 30°C for 3 days followed by cultivations at 30°C on the SD triple-dropout (TDO) medium (SD/-His/-Leu/-Trp) and the SD quadruple-dropout (QDO) medium (SD/-Ade/-His/-Leu/-Trp) to determine positive interactions.

### Pull-down and co-immunoprecipitation (Co-IP) assays

Expression vectors pMAL-c5E-NlNDUFS1, pET-28a-RRSV Pns10, pGEX-4T-3-RRSV Pns10, and pGEX-4T-3-NlPHB2 were separately transfected into *E. coli* strain BL21 (DE3) cells. Protein expressions were induced by 0.5 mM isopropyl β-D-thiogalactoside (Sigma-Aldrich, Taufkirchen, Germany) solution at 16°C for 16 hours. The expressed proteins were individually purified using Dextrin Beads (Smart-Lifesciences, Changzhou, China), High Affinity Ni-TED Resin FF (GenScript, Nanjing, China), or Glutathione Resin (GenScript, Nanjing, China). For MBP pull-down assays, the purified MBP-NlNDUFS1 or MBP-tag was co-incubated with GST-RRSV Pns10 or GST-NlPHB2 at 4°C for 4 hours, and then overnight with Dextrin Beads at 4°C. After centrifugation at 800 *g* for 2 min at 4°C, the pelleted beads were washed three times with the NETN buffer [20 mM Tris-Cl (pH 8.0), 100 mM NaCl, 0.5 mM EDTA, and 0.5% (v/v) NP-40] followed by Western blot assays using an MBP, GST or PHB2 antibody. For competitive binding pull-down assays, purified MBP-NlNDUFS1 was first mixed with GST-NlPHB2 and then mixed with gradually increased amounts of His-RRSV Pns10 followed by an overnight incubation at 4°C. The samples were then incubated individually with Dextrin Beads at 4°C for 4 hours. After centrifugation at 800 *g* for 2 min at 4°C, the beads were washed three times with the NETN buffer and analyzed through Western blot assays using an NDUFS1, RRSV Pns10 or PHB2 specific antibody.

For Co-IP assays, RRSV Pns10-GFP or GFP was co-expressed with NlNDUFS1-Flag in Sf9 cells using the pFastBac dual vector. Total protein was extracted from the Sf9 cells with a RIPA Lysis buffer (Beyotime) and incubated with anti-GFP mAb-Magnetic Beads as instructed (Medical & Biological Laboratories, Tokyo, Japan) for 4 hours at 4°C. After three washes in the RIPA Lysis buffer, the beads-bound proteins were analyzed through Western blot assays using an NDUFS1 or GFP antibody. For *in vivo* competitive binding Co-IP assays, total protein was extracted from *N. lugens* at 10 dpvf (0.554 g *N. lugens* per reaction) using the Cell lysis buffer for Western and IP (Beyotime). For semi-*in vitro* competitive binding Co-IP assays, total protein was extracted from *N. lugens* (1.88 g *N. lugens* per reaction) using the Cell lysis buffer for Western and IP (Beyotime). Purified RRSV GST-Pns10 and GST were separately added into the total protein samples and then incubated with a PHB2 antibody for 4 hours at 4°C. The above mixtures were then incubated with rProtein G agarose resin (Yeasen Biotechnology, Shanghai, China) for 4 hours at 4°C. After three washes in the Cell lysis buffer, Co-IP products were analyzed through Western blot assays using an NDUFS1 or PHB2 antibody.

### Mitochondrial membrane potential measurement and TUNEL staining

Sf9 cell mitochondrial membrane potential was determined using an assay kit with tetramethylrhodamine ethyl ester (TMRE) as instructed (Beyotime). Briefly, Sf9 cells were cultivated on cover glasses (WoHong Biotechnology, Shanghai, China) and then transfected with a Pns10-GFP or a GFP baculovirus expression vector. The transfected cells were incubated in the TMRE solution for 30 min inside an incubator. After two washes in the Sf9 cell culture medium, the cells were examined and imaged under an FV3000 Olympus confocal microscope.

Terminal deoxynucleotidyl transferase (TdT)-mediated dUTP nick-end labeling (TUNEL) was performed to visualize apoptotic cells in *N. lugens* or apoptotic Sf9 cells using One step TUNEL apoptosis assay kit (Beyotime) as instructed. Briefly, at four dpvf, intestines were collected from the assayed *N. lugens* and fixed in an Immunol staining fix solution (Beyotime). The fixed intestines were permeabilized in an Immunostaining permeabilization solution with Triton X-100 (Beyotime). After incubation in a RRSV Pns10 monoclonal antibody solution and then in a goat anti-mouse IgG-Dylight 549 solution, the intestines were incubated in a terminal deoxynucleotidyl transferase (TdT) incubation buffer for 1 hour at 37°C. The Fluorescein-dUTP signal and labeled RRSV Pns10 were examined and imaged under confocal microscope. For Sf9 cells, cells expressing either RRSV Pns10-GFP or GFP alone were fixed and permeabilized as described above for *N. lugens* intestines. The cells were then incubated in the TdT incubation buffer for 1 hour at 37°C followed by confocal microscopy observation.

### CASP3 activity, mitochondria staining, and mitochondrial complex I activity assays

A caspase 3 activity assay kit (Beyotime) was used to detect CASP3 activity in *N. lugens* and Sf9 cells. *N. lugens* (0.054 g per sample) or Sf9 cells (5 × 10^6^ cells per sample) were homogenized and incubated in 250 µL Cell lysis buffer (Beyotime) for 10 min on ice. After centrifugation at 16000 *g* for 15 min at 4°C, supernatant was collected from each sample and transferred into a new tube on ice for further use. Protein concentration of each sample was quantified using the Bradford protein assay kit (Beyotime) as instructed, followed by dilution to same concentration for subsequent enzyme activity measurements. The enzyme activity assay of CASP3 in samples was performed according to the manufacturer’s instructions, and the 405 nm absorbance was measured using a FlexStation 3 instrument (Molecular Devices, California, USA).

For mitochondria staining, Sf9 cells expressing the assayed proteins by recombinant baculovirus were grown on cover glasses, and then incubated for 45 min in a Mito-Tracker Red CMXRos solution as instructed by the manufacturer (Beyotime). After three washes in the Sf9 cell culture medium, cells were immediately examined and imaged under an FV3000 Olympus confocal microscope.

Protein concentration of each sample was determined using the BCA protein assay kit (Beyotime). Mitochondrial complex I activity was measured using a mitochondrial complex I activity assay kit (Solarbio, Beijing, China) as instructed by the manufacturer and the 340 nm absorbance of each sample was measured using the FlexStation 3 (Molecular Devices, California, USA).

### Mitochondrial isolation

Mitochondria were extracted from *N. lugens* and Sf9 cells using a Tissue mitochondria isolation kit (Beyotime) and a Cell mitochondria isolation kit (Beyotime), respectively. Briefly, *N. lugens* and Sf9 cells were homogenized in the mitochondrial separation reagent on ice and then centrifuged for 5 min at 600 *g* and 4°C. Supernatant was collected from individual samples and transferred to clean centrifuge tubes followed by 10 min centrifugation at 11000 *g* and 4˚C. The pelleted *N. lugens* or Sf9 mitochondria were analyzed through Western blot assays.

### Intracellular ATP and mitochondrial ROS level assays

Protein concentration of each sample was determined using the BCA Protein Assay Kit. The intracellular ATP level of *N. lugens* and Sf9 cells was measured using an enhanced ATP assay kit (Beyotime). The relative luminescence unit (RLU) was measured using the FlexStation 3 (Molecular Devices).

Mitochondrial ROS levels in *N. lugens* midgut cells or Sf9 cells were determined using the fluorescent probe MitoSOX as instructed (Thermo Fisher, Massachusetts, USA). *N. lugens* midgut or Sf9 cells were separately incubated for 20 min in a MitoSOX solution (5 μM) at RT and in the dark. The mitochondrial ROS level in each sample was examined and imaged under confocal microscope.

### Statistical and protein band intensity analyses

The data obtained from different assays were analyzed using the Student’s *t*-test or the one-way ANOVA followed by the Tukey’s multiple comparison test in the GraphPad Prism 8 (GraphPad Software, California, USA). The resulting statistical results are presented as the means ± SDs. For Western blot assays, the intensities of protein bands were measured using the ImageJ software as instructed (NIH, Bethesda, Maryland, USA).

## Supporting information

S1 Fig Both Pns10 1–138 and Pns10 139–297 induce apoptosis in Sf9 cells. (A) Confocal images showing the TUNEL staining signal (Cy3, red) from GFP, Pns10 1–138-GFP or Pns10 139–297-GFP expressing Sf9 apoptotic cells. Scale bar = 30 μm. (B) The percentage of apoptotic cells in GFP, Pns10 1–138-GFP or Pns10 139–297-GFP expressing Sf9 cell samples. The values are the means ± SDs (n = 7), determined using the one-way ANOVA followed by the Tukey’s multiple comparison test. ****, *P* < 0.0001. Ns, no significant statistical difference.
(DOCX)

S2 Fig Phylogenetic relationships of NDUFS1 (A) or PHB2 (B) protein between *Nilaparvata lugens* and other four species. The phylogenetic trees of NDUFS1 (A) and PHB2 (B) proteins were constructed using the maximum likelihood method with 1000 bootstraps. The tree is drawn to scale, with branch lengths measured in the number of substitutions per site. The bootstrap values are indicated adjacent to the nodes. Accession numbers of these sequences are listed in S1 and S2 Tables.
(DOCX)

S3 Fig Schematic diagram of conserved domains of NDUFS1 from *Nilaparvata lugens*, *Laodelphax striatellus*, *Spodoptera frugiperda*, *Homo sapiens* and *Mus musculus.* Amino acid position of individual domain is indicated above or under each domain box.
(DOCX)

S4 Fig The mRNA (A) and protein (B) levels of *NlPHB2* in viruliferous and nonviruliferous *N. lugens* determined by RT-qPCR and Western blot assays at 6 dpvf. The expression level of *NlActin* was used as an internal control. The values are means ± SDs (n = 23), determined by Student’s *t* test. Ns, no significant statistical difference. α-Tubulin was used as a protein loading control.
(TIF)

S5 Fig Y2H assay result showed that Pns10 1–213 did not interact with NlPHB2. *Pns10 1–213* and *NlPHB2* genes were separately cloned into the pGBKT7 and pGADT7 vectors. After co-transformation into yeast cells, cells were ten-fold diluted and plated on the SD/-Trp-Leu-His-Ade medium. The cells co-transformed with pGADT7-T and pGBKT7-p53 or pGADT7-T and pGBKT7-Lam were used as the positive and negative control.
(DOCX)

S6 Fig ATPase activity of Pns10 does not contribute to mitochondrial energy impairment or ROS accumulation in Sf9 cells. (A) A Y2H assay result showing the interaction between Pns10^K20Q^ 1–213 or Pns10^K20Q^ 1–138 and NlNDUFS1. Pns10^K20Q^ 1–213 or Pns10^K20Q^ 1–138 were cloned into the pGBKT7 vector, and NlNDUFS1 was cloned into the pGADT7 vector. After co-transformation into yeast cells, cells were ten-fold diluted and plated on the SD/-Trp-Leu-His-Ade medium. The cells co-transformed with pGADT7-T and pGBKT7-p53 or pGADT7-T and pGBKT7-Lam were used as the positive and the negative control. (B) Analysis result showing that ATP production in Pns10-GFP or Pns10^K20Q^-GFP expressing Sf9 cells was significantly reduced. The values are means ± SDs (n = 6), determined using the one-way ANOVA followed by the Tukey’s multiple comparison test. **, *P* < 0.01. Ns, no significant statistical difference. (C, D) Confocal microscopy results showing the mitochondrial ROS accumulation level (MitoSOX, red) in GFP, Pns10-GFP or Pns10^K20Q^-GFP expressing Sf9 cells. Scale bar = 10 μm. The relative strength of MitoSOX florescence signal was also measured through the ImageJ software (D). The values are the means ± SDs (n = 8), determined using the one-way ANOVA followed by the Tukey’s multiple comparison test. ****, *P* < 0.0001. Ns, no significant statistical difference.
(TIF)

S1 Table NDUFS1 protein amino acid sequence identity analysis between *Nilaparvata lugens* and other four species. (DOCX)

S2 Table PHB2 protein amino acid sequence identity analysis between *Nilaparvata lugens* and other four species. (DOCX)

S3 Table Primers used in this study. (DOCX)
