# Supplementary material for: Rice ragged stunt virus Pns10 induces mitochondrial-mediated apoptosis to promote viral infection in Nilaparvata lugens through disrupting the NlNDUFS1-NlPHB2 interaction
Source: PLoS Pathog. 2025 Aug 19;21(8):e1013415. doi: 10.1371/journal.ppat.1013415 (PMC12364342; doi:10.1371/journal.ppat.1013415)
Supplement: S1 Fig — (A) Confocal images showing the TUNEL staining signal (Cy3, red) from GFP, Pns10 1–138-GFP or Pns10 139–297-GFP expressing Sf9 apoptotic cells. Scale bar = 30 μm. (B) The percentage of apoptotic cells in GFP, Pns10 1–138-GFP or Pns10 139–297-GFP expressing Sf9 cell samples. The values are the means ± SDs (n = 7), determined using the one-way ANOVA followed by the Tukey’s multiple comparison test. ****, P < 0.0001. Ns, no significant statistical difference. (DOCX) [file ppat.1013415.s001.docx]

**
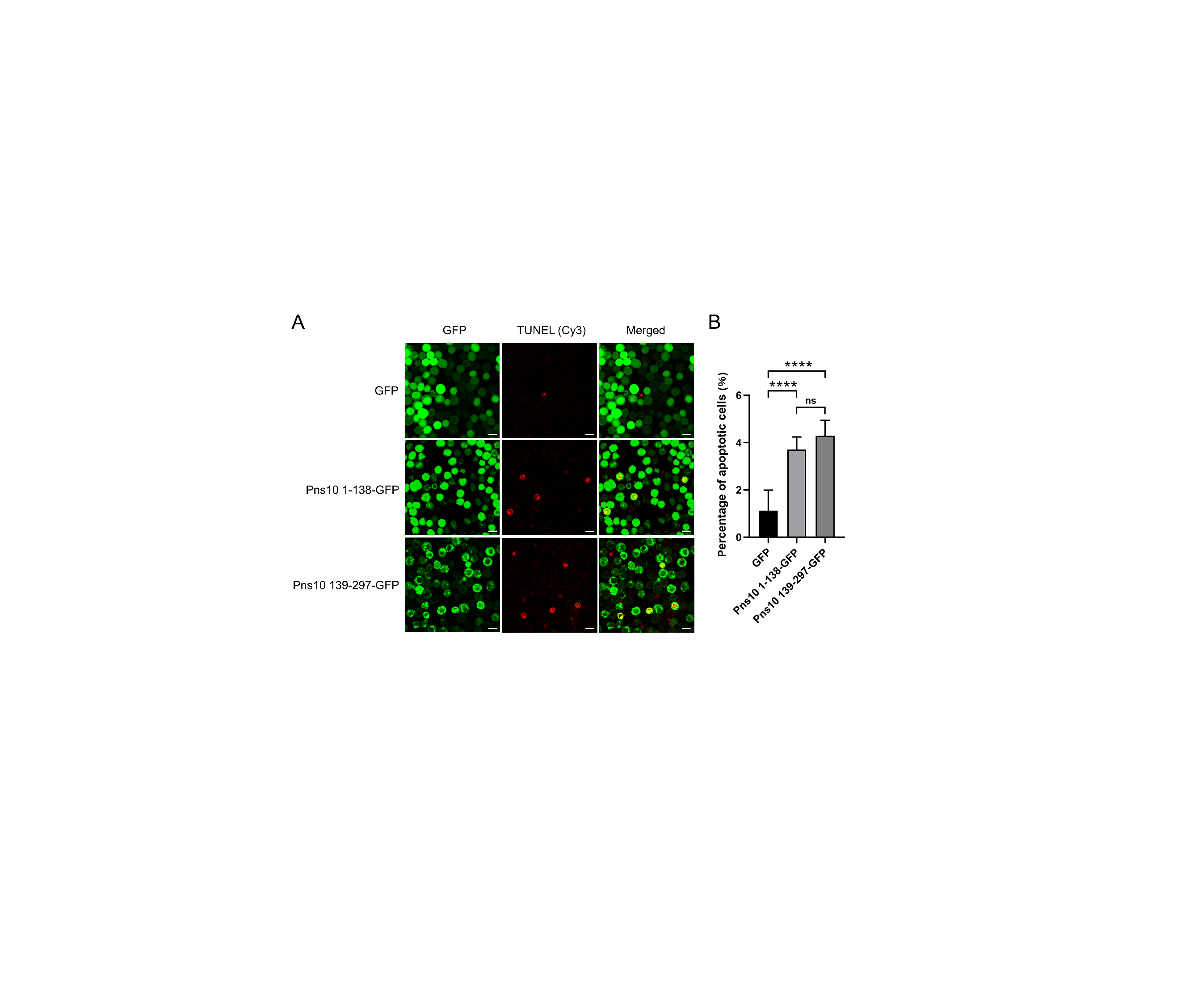
**

S1 Fig. Both Pns10 1-138 and Pns10 139-297 induce apoptosis in Sf9 cells. (A) Confocal images showing the TUNEL staining signal (Cy3, red) from GFP, Pns10 1-138-GFP or Pns10 139-297-GFP expressing Sf9 apoptotic cells. Scale bar = 30 μm. (B) The percentage of apoptotic cells in GFP, Pns10 1-138-GFP or Pns10 139-297-GFP expressing Sf9 cell samples. The values are the means ± SDs (n=7), determined using the one-way ANOVA followed by the Tukey’s multiple comparison test. ****, *P* < 0.0001. Ns, no significant statistical difference.
