# Supplementary material for: Rice ragged stunt virus Pns10 induces mitochondrial-mediated apoptosis to promote viral infection in Nilaparvata lugens through disrupting the NlNDUFS1-NlPHB2 interaction
Source: PLoS Pathog. 2025 Aug 19;21(8):e1013415. doi: 10.1371/journal.ppat.1013415 (PMC12364342; doi:10.1371/journal.ppat.1013415)
Supplement: S2 Fig — The phylogenetic trees of NDUFS1 (A) and PHB2 (B) proteins were constructed using the maximum likelihood method with 1000 bootstraps. The tree is drawn to scale, with branch lengths measured in the number of substitutions per site. The bootstrap values are indicated adjacent to the nodes. Accession numbers of these sequences are listed in S1 and S2 Tables. (DOCX) [file ppat.1013415.s002.docx]

**
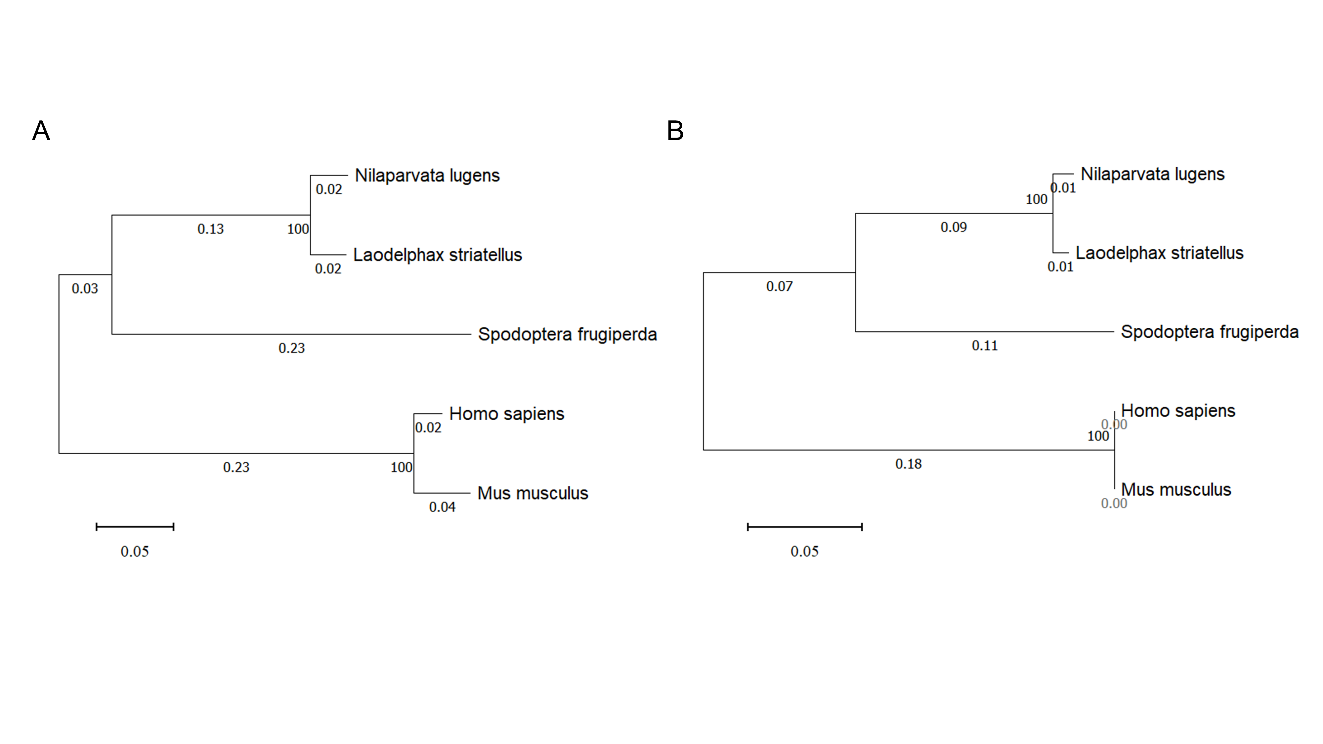
**

S2 Fig. Phylogenetic relationships of NDUFS1 (A) or PHB2 (B) protein between *Nilaparvata lugens* and other four species. The phylogenetic trees of NDUFS1 (A) and PHB2 (B) proteins were constructed using the maximum likelihood method with 1000 bootstraps. The tree is drawn to scale, with branch lengths measured in the number of substitutions per site. The bootstrap values are indicated adjacent to the nodes. Accession numbers of these sequences are listed in Table S1 and S2.
