# Supplementary material for: Rice ragged stunt virus Pns10 induces mitochondrial-mediated apoptosis to promote viral infection in Nilaparvata lugens through disrupting the NlNDUFS1-NlPHB2 interaction
Source: PLoS Pathog. 2025 Aug 19;21(8):e1013415. doi: 10.1371/journal.ppat.1013415 (PMC12364342; doi:10.1371/journal.ppat.1013415)
Supplement: S3 Fig — Amino acid position of individual domain is indicated above or under each domain box. (DOCX) [file ppat.1013415.s003.docx]

**
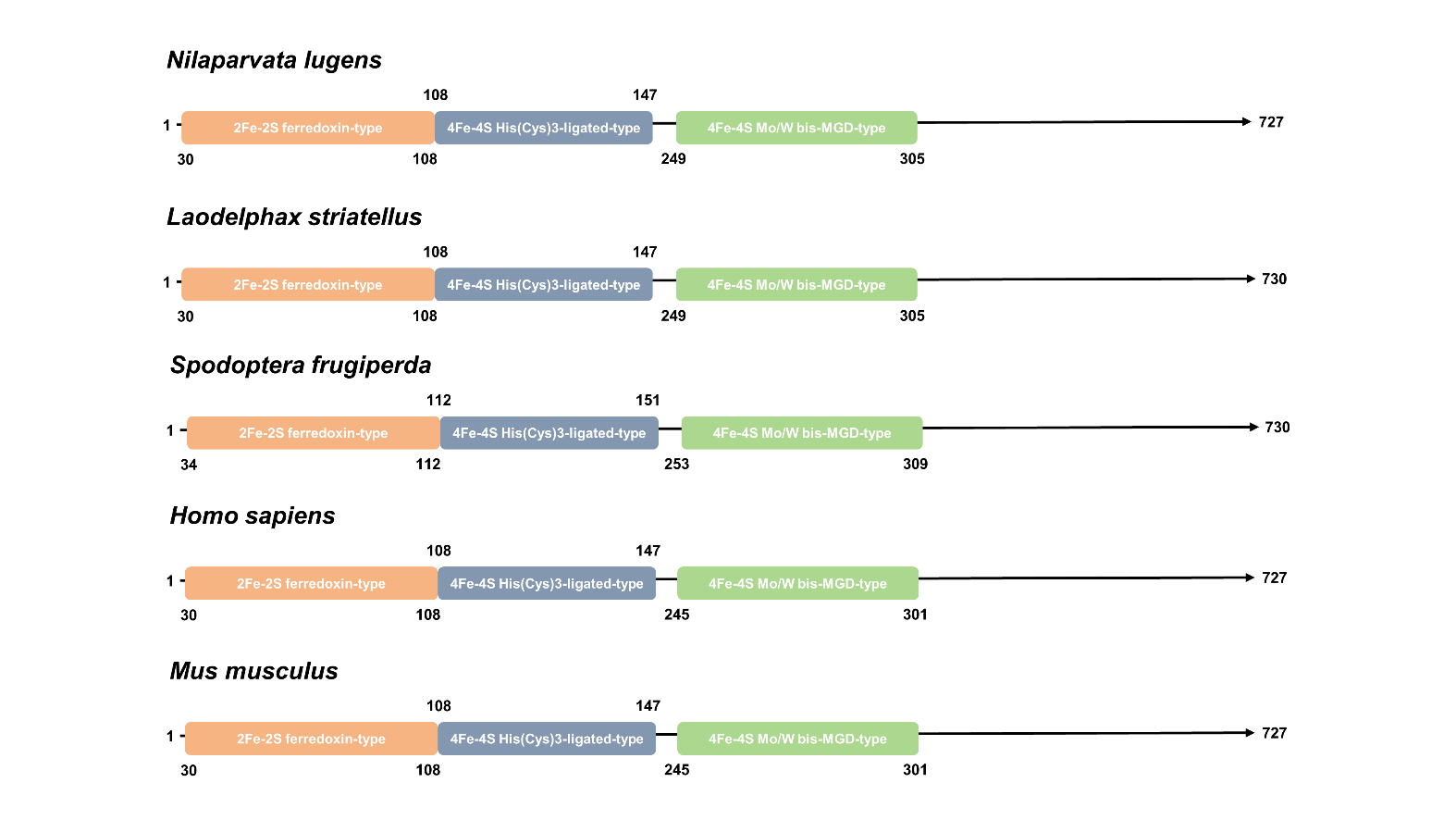
**

S3 Fig. Schematic diagram of conserved domains of NDUFS1 from *Nilaparvata lugens*, *Laodelphax striatellus*, *Spodoptera frugiperda*, *Homo sapiens* and *Mus musculus*. Amino acid position of individual domain is indicated above or under each domain box.
