# Supplementary figures and images for: Rice ragged stunt virus Pns10 induces mitochondrial-mediated apoptosis to promote viral infection in Nilaparvata lugens through disrupting the NlNDUFS1-NlPHB2 interaction
Source: PLoS Pathog. 2025 Aug 19;21(8):e1013415. doi: 10.1371/journal.ppat.1013415 (PMC12364342; doi:10.1371/journal.ppat.1013415)

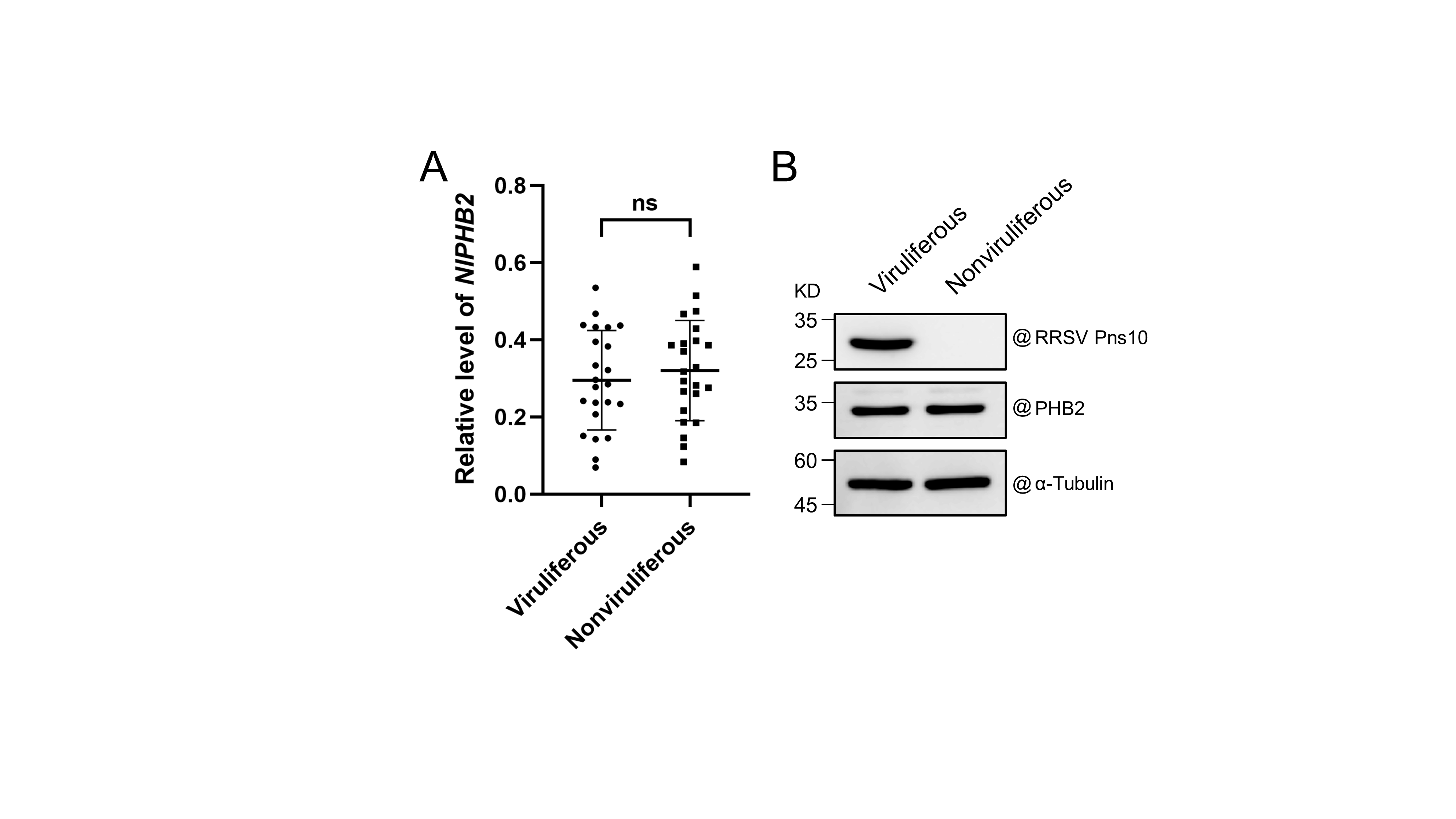

Supplement: S4 Fig — The expression level of NlActin was used as an internal control. The values are means ± SDs (n = 23), determined by Student’s t test. Ns, no significant statistical difference. α-Tubulin was used as a protein loading control. (TIF) [file ppat.1013415.s004.tif]

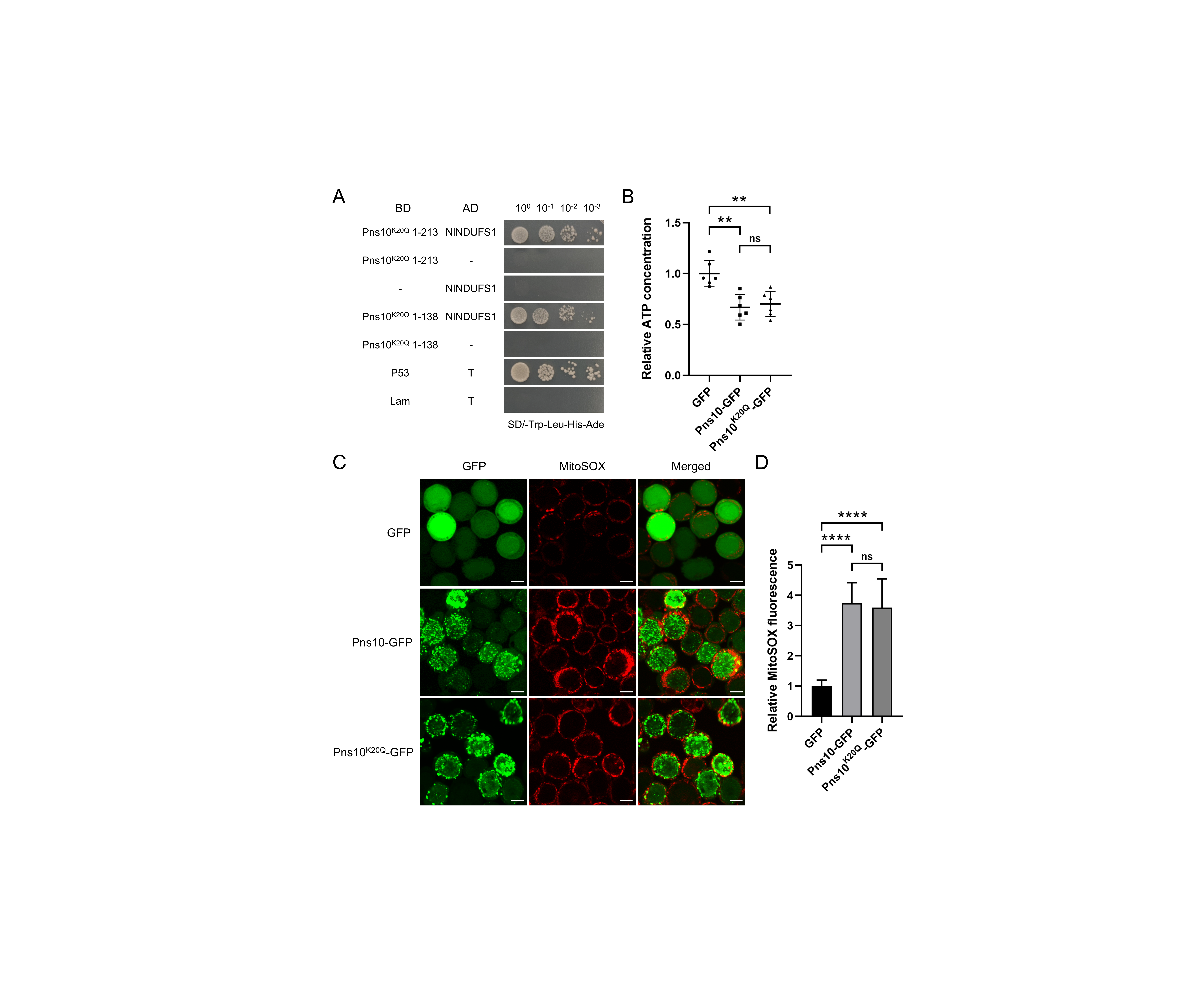

Supplement: S6 Fig — (A) A Y2H assay result showing the interaction between Pns10K20Q 1–213 or Pns10K20Q 1–138 and NlNDUFS1. Pns10K20Q 1–213 or Pns10K20Q 1–138 were cloned into the pGBKT7 vector, and NlNDUFS1 was cloned into the pGADT7 vector. After co-transformation into yeast cells, cells were ten-fold diluted and plated on the SD/-Trp-Leu-His-Ade medium. The cells co-transformed with pGADT7-T and pGBKT7-p53 or pGADT7-T and pGBKT7-Lam were used as the positive and the negative control. (B) Analysis result showing that ATP production in Pns10-GFP or Pns10K20Q-GFP expressing Sf9 cells was significantly reduced. The values are means ± SDs (n = 6), determined using the one-way ANOVA followed by the Tukey’s multiple comparison test. **, P < 0.01. Ns, no significant statistical difference. (C, D) Confocal microscopy results showing the mitochondrial ROS accumulation level (MitoSOX, red) in GFP, Pns10-GFP or Pns10K20Q-GFP expressing Sf9 cells. Scale bar = 10 μm. The relative strength of MitoSOX florescence signal was also measured through the ImageJ software (D). The values are the means ± SDs (n = 8), determined using the one-way ANOVA followed by the Tukey’s multiple comparison test. ****, P < 0.0001. Ns, no significant statistical difference. (TIF) [file ppat.1013415.s006.tif]
