# Supplementary material for: Rice ragged stunt virus Pns10 induces mitochondrial-mediated apoptosis to promote viral infection in Nilaparvata lugens through disrupting the NlNDUFS1-NlPHB2 interaction
Source: PLoS Pathog. 2025 Aug 19;21(8):e1013415. doi: 10.1371/journal.ppat.1013415 (PMC12364342; doi:10.1371/journal.ppat.1013415)
Supplement: S5 Fig — Pns10 1–213 and NlPHB2 genes were separately cloned into the pGBKT7 and pGADT7 vectors. After co-transformation into yeast cells, cells were ten-fold diluted and plated on the SD/-Trp-Leu-His-Ade medium. The cells co-transformed with pGADT7-T and pGBKT7-p53 or pGADT7-T and pGBKT7-Lam were used as the positive and negative control. (DOCX) [file ppat.1013415.s005.docx]

**
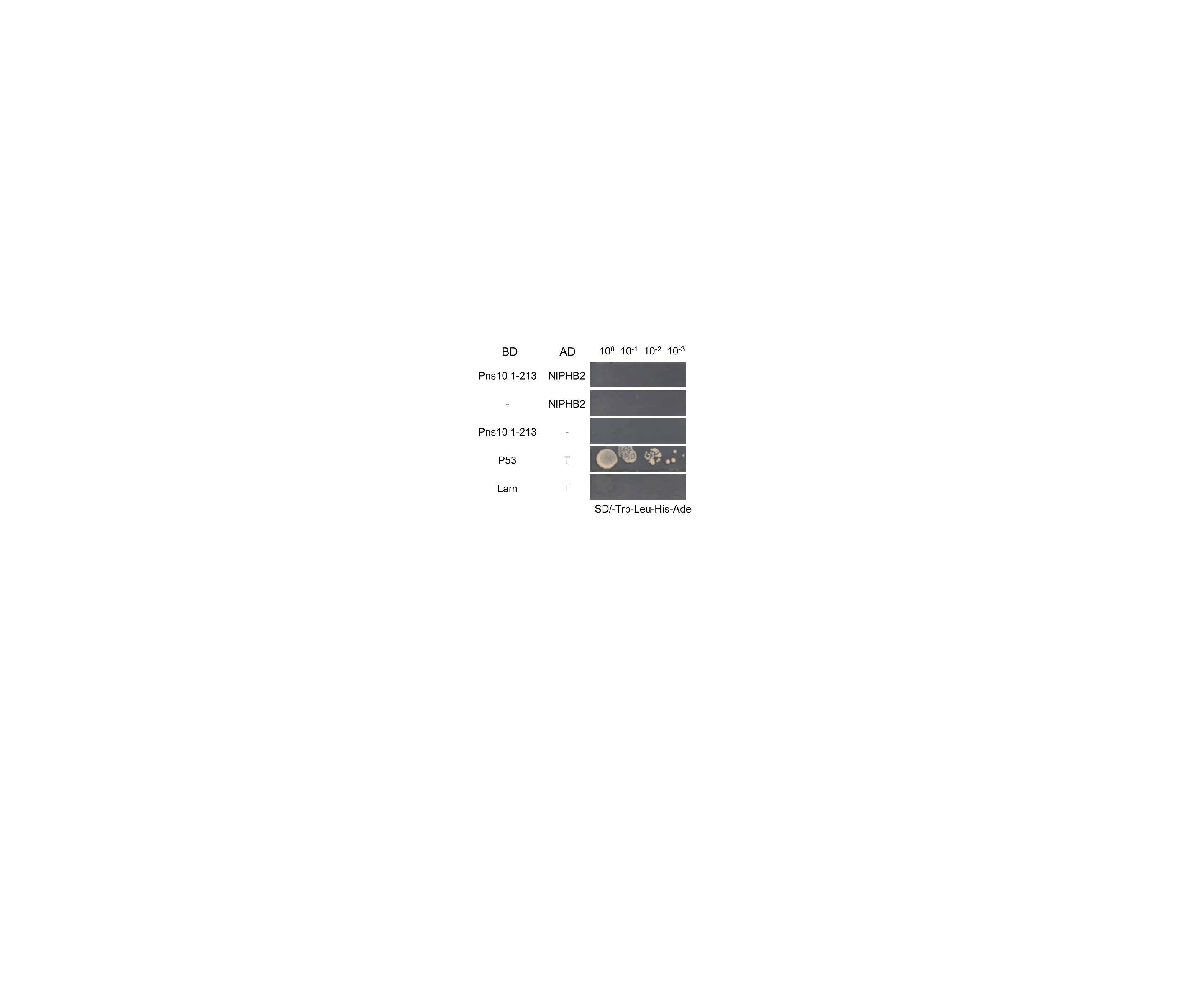
**

S5 Fig. Y2H assay result showed that Pns10 1-213 did not interact with NlPHB2. *Pns10 1-213* and *NlPHB2* genes were separately cloned into the pGBKT7 and pGADT7 vectors. After co-transformation into yeast cells, cells were ten-fold diluted and plated on the SD/-Trp-Leu-His-Ade medium. The cells co-transformed with pGADT7-T and pGBKT7-p53 or pGADT7-T and pGBKT7-Lam were used as the positive and negative control.
