# Supplementary material for: Rice ragged stunt virus Pns10 induces mitochondrial-mediated apoptosis to promote viral infection in Nilaparvata lugens through disrupting the NlNDUFS1-NlPHB2 interaction
Source: PLoS Pathog. 2025 Aug 19;21(8):e1013415. doi: 10.1371/journal.ppat.1013415 (PMC12364342; doi:10.1371/journal.ppat.1013415)
Supplement: S1 Table — (DOCX) [file ppat.1013415.s007.docx]

S1 Table. NDUFS1 protein amino acid sequence identity analysis between *Nilaparvata lugens* and other four species.

| **Species** | **Accession numbers** | **Amino acid identities (%)** |
| --- | --- | --- |
| *Laodelphax striatellus* | RZF43427.1 | 95.2 |
| *Spodoptera frugiperda* | XP_035443662.2 | 70.4 |
| *Homo sapiens* | NP_001186913.1 | 68.7 |
| *Mus musculus* | AAH06660.1 | 68.0 |

Red number indicates the highest similarity.
