# Supplementary material for: Rice ragged stunt virus Pns10 induces mitochondrial-mediated apoptosis to promote viral infection in Nilaparvata lugens through disrupting the NlNDUFS1-NlPHB2 interaction
Source: PLoS Pathog. 2025 Aug 19;21(8):e1013415. doi: 10.1371/journal.ppat.1013415 (PMC12364342; doi:10.1371/journal.ppat.1013415)
Supplement: S2 Table — (DOCX) [file ppat.1013415.s008.docx]

S2 Table. PHB2 protein amino acid sequence identity analysis between *Nilaparvata lugens* and other four species.

| **Species** | **Accession numbers** | **Amino acid identities (%)** |
| --- | --- | --- |
| *Laodelphax striatellus* | RZF43489.1 | 96.9 |
| *Spodoptera frugiperda* | XP_050549974.1 | 82.9 |
| *Homo sapiens* | NP_001138303.1 | 72.1 |
| *Mus musculus* | NP_031557.2 | 72.1 |

Red number indicates the highest similarity.
