# Supplementary material for: Rice ragged stunt virus Pns10 induces mitochondrial-mediated apoptosis to promote viral infection in Nilaparvata lugens through disrupting the NlNDUFS1-NlPHB2 interaction
Source: PLoS Pathog. 2025 Aug 19;21(8):e1013415. doi: 10.1371/journal.ppat.1013415 (PMC12364342; doi:10.1371/journal.ppat.1013415)
Supplement: S3 Table — (DOCX) [file ppat.1013415.s009.docx]

S3 Table. Primers used in this study.

| **Note** | **Primer name** | **Sequence (5’-3’)** |
| --- | --- | --- |
| RT-qPCR | *NlCaspase1a*-qPCR-F | GAGGCAAAGAGTCCAGTAGCA |
|  | *NlCaspase1a*-qPCR-R | AACCGAGTCTCGTCAAAGTGT |
|  | *NlCaspase8*-qPCR-F | GCAATCCCGAGGATCTAGGC |
|  | *NlCaspase8*-qPCR-R | CCTGCAACCTCTCAACGTCT |
|  | *NlCaspaseNc*-qPCR-F | ACCATGGAAAACGTGTTGAAAAA |
|  | *NlCaspaseNc*-qPCR-R | AACTAGTTGATAGACCAGGTTCAA |
|  | *NlNDUFS1*-qPCR-F | actactgttcttcaggcggc |
|  | *NlNDUFS1*-qPCR-R | ccggctttggtgacttttcg |
|  | *RRSV-P8*-qPCR-F | tcgcacgagacgttctttga |
|  | *RRSV-P8*-qPCR-R | gataccgtttaggcgctgga |
|  | *NlPHB2*-qPCR-F | aagcattgctcgcacgattg |
|  | *NlPHB2*-qPCR-R | tcgacgtccaggagctatgt |
|  | *NlACTIN*-qPCR-F | gatcaagatcattgccccgc |
|  | *NlACTIN*-qPCR-R | gtgcacaattgatgggccag |
| dsRNA | *NlCaspase1a*-dsRNA-F | TAATACGACTCACTATAGGGGGAGGATCATTCCGACTTCG |
|  | *NlCaspase1a*-dsRNA-R | TAATACGACTCACTATAGGGAAGAGGCGGGTGAGCATGAA |
|  | *NlCaspase8*-dsRNA-F | TAATACGACTCACTATAGGGATTGTTGAGAATGACGTGCC |
|  | *NlCaspase8*-dsRNA-R | TAATACGACTCACTATAGGGAGAAAGTCATCGTTGTGTCC |
|  | *NlCaspaseNc*-dsRNA-F | TAATACGACTCACTATAGGGGTTTCGTTGGGCAACTGGTA |
|  | *NlCaspaseNc*-dsRNA-R | TAATACGACTCACTATAGGGTCGCCATTGTCAGGAGTGTT |
|  | *NlNDUFS1*-dsRNA-F | TAATACGACTCACTATAGGGatagatttgtgcccggttgg |
|  | *NlNDUFS1*-dsRNA-R | TAATACGACTCACTATAGGGTCGTGCCAACCAACAGAATC |
|  | *NlPHB2*-dsRNA-F | TAATACGACTCACTATAGGGATTTGGAGGAAGTCCGAAAG |
|  | *NlPHB2*-dsRNA-R | TAATACGACTCACTATAGGGCAACTGCAGCGGTATATTCT |
|  | *GFP*-dsRNA-F | TAATACGACTCACTATAGGGCCAGTATAAAGAAGAACAGCC |
|  | *GFP*-dsRNA-R | TAATACGACTCACTATAGGGCCTCTTCATGGTGTTCCTGG |
| Yeast Two-Hybrid | BD-*RRSV-Pns10*-*1*-*298*-F | ATGGCCATGGAGGCCGAATTCATGCCTTTCGTGCAATTCCC |
|  | BD-*RRSV-Pns10*-*1*-*298*-R | CCGCTGCAGGTCGACGGATCCCTACTCTGCGTCATCACCAA |
|  | BD-*RRSV-Pns10*-*1*-*213*-F | ATGGCCATGGAGGCCGAATTCATGCCTTTCGTGCAATTCCC |
|  | BD-*RRSV-Pns10*-*1*-*213*-R | CCGCTGCAGGTCGACGGATCCTCCAAGAGTAGTTGGTTCACC |
|  | BD-*RRSV-Pns10*-*1*-*138*-F | ATGGCCATGGAGGCCGAATTCATGCCTTTCGTGCAATTCCC |
|  | BD-*RRSV-Pns10*-*1*-*138*-R | CCGCTGCAGGTCGACGGATCCCTACACAGTTTCAAGTCCTTGTG |
|  | BD-*RRSV-Pns10*-*139*-*298*-F | ATGGCCATGGAGGCCGAATTCATGCAAATGATGGATTATATTC |
|  | BD-*RRSV-Pns10*-*139*-*298*-R | CCGCTGCAGGTCGACGGATCCCTACTCTGCGTCATCACCAA |
|  | BD-*RRSV-Pns10*-*66*-*298*-F | ATGGCCATGGAGGCCGAATTCTTTGATGAACCATGTTTCGTTC |
|  | BD-*RRSV-Pns10*-*66*-*298*-R | CCGCTGCAGGTCGACGGATCCCTACTCTGCGTCATCACCAA |
|  | AD-*NlNDUFS1*-F | GCCATGGAGGCCAGTGAATTCATGCTTCGCACCCCTTTGCA |
|  | AD-*NlNDUFS1*-R | CAGCTCGAGCTCGATGGATCCCTAGGCTGATTGCGCCTCGTAA |
|  | AD-*NlNDUFS1*-*1*-*305*-F | GCCATGGAGGCCAGTGAATTCATGCTTCGCACCCCTTTGCA |
|  | AD-*NlNDUFS1*-*1*-*305*-R | CAGCTCGAGCTCGATGGATCCTCGCTGCCGCTTCAGGCCGT |
|  | AD-*NlNDUFS1*-*306*-*728*-F | GCCATGGAGGCCAGTGAATTCCTCATCTCGCCGATGCTGAA |
|  | AD-*NlNDUFS1*-*306*-*728*-R | CAGCTCGAGCTCGATGGATCCCTAGGCTGATTGCGCCTCGT |
|  | BD-*NlPHB2*-F | ATGGCCATGGAGGCCGAATTCATGTCGCAAAGTAAATTAAATGAT |
|  | BD-*NlPHB2*-R | CCGCTGCAGGTCGACGGATCCTCATTTAGCTACAAGCTGGGC |
| Prokaryotic expression | GST-*RRSV*-*Pns10*-F | GATCTGGTTCCGCGTGGATCCATGCCTTTCGTGCAATTCCC |
|  | GST-*RRSV*-*Pns10*-R | GATGCGGCCGCTCGAGTCGACCTACTCTGCGTCATCACCAAA |
|  | MBP-*NlNDUFS1*-F | TATCGGAATTAATTCGGATCCGATGCTTCGCACCCCTTTGC |
|  | MBP-*NlNDUFS1*-R | TGCGGCCGCAAGCTTGTCGACCTAGGCTGATTGCGCCTCGT |
|  | GST-*NlPHB2*-F | GATCTGGTTCCGCGTGGATCCATGTCGCAAAGTAAATTAAATGAT |
|  | GST-*NlPHB2*-R | GATGCGGCCGCTCGAGTCGACTCATTTAGCTACAAGCTGGGC |
|  | His-*RRSV*-*Pns10*-F | CAGCAAATGGGTCGCGGATCCATGCCTTTCGTGCAATTCCC |
|  | His-*RRSV*-*Pns10*-R | TGCGGCCGCAAGCTTGTCGACCTCTGCGTCATCACCAAAGT |
| Baculovirus expression | pFast-*GFP*-*RRSV*-*Pns10*-F | TTTCAAGGCGCCATGGGATCCATGCCTTTCGTGCAATTCCC |
|  | pFast-*GFP*-*RRSV*-*Pns10*-R | GCCGCGACTAGTGAGCTCGTCGACTTACTTGTACAGCT |
|  | pFast-*GFP*-*NlNDUFS1*-F | TTTCAAGGCGCCATGGGATCCATGCTTCGCACCCCTTTGCA |
|  | pFast-*GFP*-*NlNDUFS1*-R | GCCGCGACTAGTGAGCTCGTCGACTTACTTGTACAGCT |
|  | pFastDual- *NlNDUFS1*-CFP-F | CCCACCATCGGGCGCGGATCCATGCTTCGCACCCCTTTGCA |
|  | pFastDual- *NlNDUFS1*-CFP-R | GCGACTAGTGAGCTCGTCGACTTACTTGTACAGCTCGTCCATGC |
|  | pFastDual-*GFP*-F | ACCATGGCTCGAGATCCCGGGTTACTTGTACAGCTCGTCCATG |
|  | pFastDual-*GFP*-R | ACGAAGACTTGATCACCCGGGATGGTGAGCAAGGGCGAGGA |
|  | pFastDual-*Pns10*-*GFP*-F | ACCATGGCTCGAGATCCCGGGTTACTTGTACAGCTCGTCCATG |
|  | pFastDual-*Pns10*-*GFP*-R | ACGAAGACTTGATCACCCGGGATGCCTTTCGTGCAATTCCC |
|  | pFastDual-*NlNDUFS1*-*Flag*-F | CCCACCATCGGGCGCGGATCCATGCTTCGCACCCCTTTGCA |
|  | pFastDual-*NlNDUFS1*-*Flag*-R | GCGACTAGTGAGCTCGTCGACTCACTTATCGTCATCGTCTTTGT |
|  | pFastDual-*NlNDUFS1*-*RFP*-F | CCCACCATCGGGCGCGGATCCATGCTTCGCACCCCTTTGCA |
|  | pFastDual-*NlNDUFS1*-*RFP*-R | GCGACTAGTGAGCTCGTCGACTTACAGGAACAGGTGGTGGC |
